# Genetic association of antinuclear antibodies with HLA in JIA patients: a Swedish cohort study

**DOI:** 10.1186/s12969-024-01017-8

**Published:** 2024-08-26

**Authors:** Raya Saleh, Erik Sundberg, Mia Olsson, Katarina Tengvall, Lars Alfredsson, Ingrid Kockum, Leonid Padyukov, Helena Erlandsson Harris

**Affiliations:** 1 Department of Medicine Solna, Division of Rheumatology, Karolinska Institutet, and Karolinska University Hospital, Stockholm, Sweden; 2 Center for Molecular Medicine, Karolinska Institutet, Stockholm, Sweden; 3 Pediatric Rheumatology unit, Astrid Lindgren’s Children’s Hospital, Karolinska University Hospital, Stockholm, Sweden; 4 Department of Clinical Neuroscience, Neuroimmunology Unit, The Karolinska Neuroimmunology & Multiple Sclerosis Centre, Karolinska Institutet, Stockholm, Sweden; 5 Institute for Environmental Medicine, Karolinska Institutet, Stockholm, Sweden; 6 Department Clinical Medicine, Broegelmann Research Laboratory, University of Bergen, Bergen, Norway

**Keywords:** Juvenile idiopathic arthritis (JIA), Genetic predisposition, Human leukocyte antigen (HLA) alleles, Major histocompatibility complex (MHC), Genome-wide association study (GWAS), Autoimmune disease, ANA positivity, JIA subtypes, Pediatric rheumatology

## Abstract

**Background:**

Juvenile Idiopathic Arthritis (JIA) is a complex autoimmune disease and the most common chronic rheumatological disease affecting children under the age of 16. The etiology of JIA remains poorly understood, but evidence suggests a significant genetic predisposition.

**Methods:**

We analyzed a Swedish cohort of 329 JIA patients and 728 healthy adult controls using the Illumina OmniExpress array for genotyping. HLA alleles were imputed from GWAS data using the SNP2HLA algorithm.

**Results:**

Case–control analysis yielded 12 SNPs with genome-wide significant association to JIA, all located on chromosome 6 within the MHC class II gene region. Notably, the top SNP (rs28421666) was located adjacent to *HLA-DQA1* and *HLA-DRB1*. HLA-DRB1*08:01, HLA-DQA1*04:01, and HLA-DQB1*04:02 were the haplotypes most strongly associated with an increased risk of JIA in the overall cohort. When analyzing disease specific subtypes, these alleles were associated with oligoarthritis and RF-negative polyarthritis. Within the complex linkage disequilibrium of the *HLA-DRB1-DQA1-DQB1* haplotype, our analysis suggests that HLA-DRB1*08 might be the primary allele linked to JIA susceptibility. The HLA-DRB1*11 allele group was also independently associated with JIA and specifically enriched in the oligoarthritis patient group. Additionally, our study revealed a significant correlation between antinuclear antibody (ANA) positivity and specific HLA alleles. The ANA-positive JIA group showed stronger associations with the *HLA-DRB1-DQA1-DQB1* haplotype, HLA-DRB1*11, and HLA-DPB1*02, suggesting a potential connection between genetic factors and ANA production in JIA. Furthermore, logistic regression analysis reaffirmed the effects of HLA alleles, female sex, and lower age at onset on ANA positivity.

**Conclusions:**

This study identified distinct genetic associations between HLA alleles and JIA subtypes, particularly in ANA-positive patients. These findings contribute to a better understanding of the genetic basis of JIA and provide insights into the genetic control of autoantibody production in ANA-positive JIA patients. This may inform future classification and personalized treatment approaches for JIA, ultimately improving patient outcomes and management of this disease.

**Supplementary Information:**

The online version contains supplementary material available at 10.1186/s12969-024-01017-8.

## Background

Juvenile idiopathic arthritis (JIA) is an umbrella term used to describe a heterogeneous group of conditions characterized by arthritis of unknown origin that develops in children under the age of 16 and lasts for at least six weeks [1]. JIA is the most common chronic rheumatological disease among children, with an incidence rate of 15 per 100,000 children/year in the Nordic countries [2].

Clinically, the disease is grouped into subtypes that are considered to be complex phenotypes, influenced by both genetic and environmental factors, potentially varying among different ethnic groups [3]. According to International League of Associations for Rheumatology (ILAR) classification criteria, JIA is divided into seven subtypes based on clinical and laboratory findings during the first 6 months of disease: oligoarthritis, polyarthritis which is further divided into patients based on the presence of rheumatoid factor (RF) autoantibodies: either RF-negative polyarthritis or RF-positive polyarthritis, psoriatic arthritis, enthesitis-related arthritis (ERA), systemic JIA and undifferentiated arthritis for cases that fit into no category or into more than one category [1].

Although the cause of the disease remains unknown, there is substantial evidence of genetic predisposition to JIA. Disease concordance for JIA in monozygotic twins is 25–40% [4], while siblings have an 11.6-fold increase in the risk of developing JIA and first cousins have a 5.6-fold increased risk [5]. Furthermore, the prevalence of other autoimmune diseases, particularly rheumatoid arthritis (RA), is increased in the relatives of patients with JIA and overlap in genetic susceptibility loci for these two diseases has been shown [6].

Several susceptibility loci have been reported in JIA [7–10]. Similar to many other autoimmune disorders, the most significant identified genetic susceptibility factors for JIA are linked to the human leukocyte antigen (HLA) locus. The HLA class I and class II haplotypes associated with the risk of JIA exhibit variations among its different subtypes [11, 12]. It was estimated that approximately 13% of JIA risk can be attributed to the HLA region, while the top 27 non-HLA loci account for about 6% of the risk [9].

Since a notable part of JIA's hereditary risk remains unclear, it is crucial to conduct comparative investigations across diverse populations to gain insight into the disease's heterogeneity and unveil critical pathways associated with it. Conversely, in the context of autoantibodies in JIA, it is worth noting that apart from Rheumatoid Factor (RF), which is used as a biomarker for diagnosis of JIA, information about other known autoantibodies, such as antibodies against citrullinated peptide/protein antigens (ACPA) and anti-nuclear antibodies (ANA), has not been utilised for classification of JIA subtypes. Traditionally, ANA has not been used as a diagnostic marker for JIA, but rather as a risk indicator associated with the most common extra-articular manifestation in JIA—chronic asymptomatic uveitis [13, 14].

This study aimed to accomplish three main objectives: first, to identify genetic risk factors associated with JIA in a Swedish cohort through a Genome-Wide Association Study (GWAS) analysis; second, to enhance the understanding of HLA associations by imputing classical HLA alleles within all JIA patients as one group and within specific subtypes; and third, to investigate the association between these HLA alleles and autoantibodies such as ANA, RF, ACPA, along with patient clinical parameters.

## Materials and methods

### Subjects

Blood samples (EDTA blood) were collected from patients at the Astrid Lindgren Children's Hospital in Stockholm, Sweden, between 2010 and 2017, as part of the Juvenile Arthritis BioBank (JABBA). Patients were enrolled and diagnosed according to the International League of Associations for Rheumatology (ILAR) criteria [1]. From the Swedish Pediatric Rheumatology Quality Registry or medical records, age at onset and autoantibody status for RF and ANA were extracted. The genotyping data from randomly selected healthy controls that had been recruited between 2005 and 2011 in a Swedish national population registry, was used in this study; this data had been generated as part of a nationwide epidemiological study, the Epidemiological Investigation of Multiple Sclerosis (EIMS) [15]. Informed consent was given by all study participants. Ethical approval for JIA patients and control subjects was obtained from the Regional Review Board in Stockholm (2009/1139–4, 2010/165–31/2, 04–252/1–4).

### Anti–CCP antibody analysis

Antibodies against cyclic citrullinated peptide/proteins (anti-CCP) were measured using the anti-CCP2 ELISA kit, Immunoscan CCPlus® CCP2 ELISA (Euro-Diagnostica AB, Malmö, Sweden). IgG anti-CCP positivity was determined according to the manufacturer’s instructions, and cutoff for positivity was set at 25 AU/mL. These data was available for 300 of the JIA patients.

### ANA and RF analysis

ANA and RF were measured by a certified clinical laboratory at Karolinska University Hospital as part of the clinical diagnosis, and information regarding seropositivity for each antibody was retrieved from medical records. RF-IgM was measured with FEIA method (Thermo Fisher/Phadia). The cutoff corresponds to 5% positivity in healthy adult blood donors and follows the ACR/EULAR guidelines for RA. ANA is measured at the disease onset by Indirect immunofluorescence (IIF) (Hep-2 cells) and cutoff is a titer of ≥ 1:320 serum dilution, which corresponds to 3–5% positivity on healthy adult blood donors (in accordance with the Swedish laboratory guidelines for ANA). ANA data was available for 297 JIA patients and RF data was available for 197 JIA patients.

### SNP genotyping and quality control

DNA extraction was carried out using the salting-out method. 335 JIA patients were genotyped using Illumina assay InfiniumOmniExpressExome-8v1-4_A (Illumina Inc). Healthy controls had previously been genotyped using the Illumina OmniExpress assay [16]. Genotypes from JIA patients and controls were merged into one dataset with approximately 597 K SNPs overlapping between the two assays for the 22 autosomes. We applied genotype filtering quality control, retaining individuals with call rate ≥ 95%, SNPs with < 5% missingness, Hardy–Weinberg equilibrium exact p ≥ 10^–3^ and minor allele frequency (MAF) > 0.01. Samples were also checked for inconsistencies between recorded and genotype-inferred gender, duplicates, and first- and second-degree relatives, resulting in a final dataset comprising 329 JIA patients, 748 controls, and ~ 589 K SNPs for the analyses.

### HLA imputation

Classical HLA alleles (*HLA-A*, *HLA-B*, *HLA-C*, *HLA-DRB1*, *HLA-DQA1*, *HLA-DQB1*, *HLA-DPA1* and *HLA-DPB1)* at four digit resolution were imputed using SNP2HLA (V.1.0) (http://www.broadinstitute.org/mpg/snp2hla/) and Type 1 Diabetes Genetics Consortium (T1DGC) reference panel (*n* = 5225 individuals with European ancestry) having genotype data of 7,135 SNPs within the MHC region assayed with Illumina Immunochip platform [17]. Post-imputation quality control was performed by removing rare variants with a MAF < 0.01, and variants with low quality *r*^2^ < 0.8 for correlation of genotyped variants with genotypes after imputation.

### Association testing and statistical analysis

To assess the dataset for potential systematic over-inflation due to population stratification, the genomic control inflation factor (*λ*_GC_) was calculated (*λ*_GC_ = 1.09) and Quantile–quantile (Q–Q) plots were generated (*Supplementary Fig. 1A*). Principal-component analysis (PCA) was performed using PLINK on a subset of SNPs, removing SNPs in known regions of high linkage disequilibrium (LD), with MAF < 0.05, and pruned for LD between markers using a sliding window approach based on *r*^2^ = 0.2. To identify the top associated markers (SNPs and two-digit HLA alleles) using a case–control design, association analysis was performed using logistic regression in PLINK (v 1.9) based on an additive genetic model. Sex and the first ten principal components were added as covariates to correct for any systematic errors and population stratification (*λ*_GC_ after correction = 1.02) (*Supplementary Fig. 1B*). *P* values obtained were reported both as non-adjusted and adjusted for multiple testing by estimating the false discovery rate (FDR) using the Benjamini–Hochberg method. SNPs with FDR < 0.05 were considered significant. The standard threshold for genome-wide significant association considered at *p* < 5 × 10^−8^. Regional plots were generated using LocusZoom [18]. To investigate subtype-specific effects, data for each subtype were compared separately against the data from the same control group. Disease association heterogeneity was tested by searching for significant differences in SNPs/HLA alleles frequency in different subtypes. Furthermore, to identify additional independent associations within the HLA region, conditional analysis on the primary associated variant was performed using PLINK [19], by adding the most significantly associated marker as a covariate to the logistic regression model. This analysis was continued in a forward stepwise procedure until no variant remained statistically significant. For correlation analysis, Phi coefficient for binary variables was calculated in R, and *P* values were calculated using chi-square test, with *P* < 0.05 considered significant. For studying association of ANA with predisposing alleles and disease parameter phenotypes, the Mann–Whitney test was used for comparison of age, and Fisher's exact test was used for analysis of other parameters. *P* values were adjusted for multiple comparisons using the Bonferroni method. Multiple logistic regression analysis was performed to ascertain the effects of independently significant variables on the likelihood of ANA status. Odds ratios and corresponding 95% confidence intervals were calculated. A *P* value of less than 0.05 was considered statistically significance. Data were analyzed using GraphPad Prism version 9.5.1.

## Results

### Study population

Our study population consisted of 329 individuals with JIA diagnosed at Karolinska University Hospital. The distribution of JIA subtypes in the dataset was as follows: oligoarthritis *n* = 161 (49%), RF-negative polyarthritis *n* = 87 (26.4%), systemic *n* = 24 (7.3%), ERA *n* = 22 (6.7%), psoriatic *n* = 19 (5.8%), RF-positive polyarthritis *n* = 12 (3.6%), and undifferentiated *n* = 4, (1.2%). The mean age at diagnosis was 79 months, and 229 patients (70%) were females. The healthy control population consisted of 748 healthy adults, of whom 568 (76%) were females. The average age was 43.5 years, and the median age was 42.7 years. An overview of the study population is provided in *Supplementary Table 1*.

### Genome-wide association analysis of JIA

**Fig. 1:**
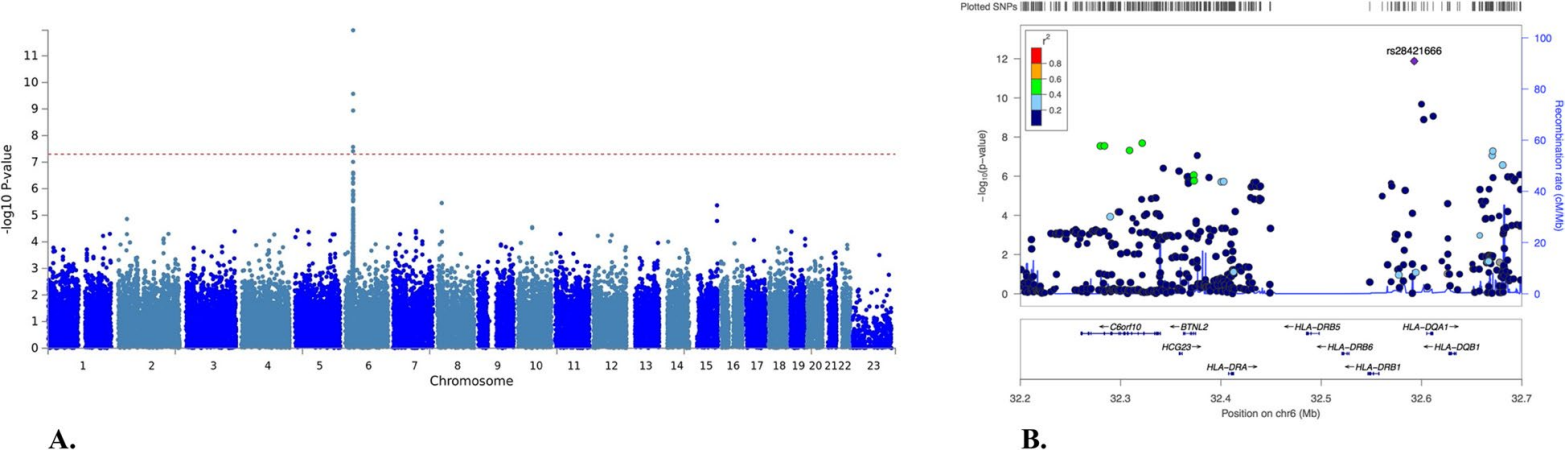
**A** Manhattan plot of the genome-wide association of 329 JIA patients compared to 748 controls. The − log_10_ *P* values for each SNP in the association tests are shown on the y axis and the chromosomes are ordered on the x axis. Twelve genetic loci in the HLA region surpassed the genome-wide significance threshold (*P* < 5 × 10^–8^ =  − log10(P) > 7.3; indicated by the red dotted line). **B** Regional association plot of the genome‐wide association of JIA with SNPs at chromosome 6 (Chr6). The top associated SNP (rs28421666) at the HLA locus is located in between *HLA-DRB1* and *HLA-DQA1.* The level of linkage disequilibrium between SNPs in the zoomed in regions of chromosome 6 is indicated by r.^2^

We first performed GWAS using genotyping data from JIA individuals and healthy controls. Case–control association analysis, adjusted for sex and the first 10 principal components, identified 12 SNPs associated with JIA. All SNPs exceeding the threshold for genome-wide significance were located on chromosome 6 (*P* < 5 × 10^−8^, FDR < 0.05). A Manhattan plot of the GWAS data with *P*-values of all tested SNPs is shown in Fig. 1A. All significantly associated SNPs on chromosome 6 were in the MHC class II gene region. The top SNP (rs28421666, *P* = 1.325E-12, FDR = 7.81E-07, OR:3.51) was in a non-coding region, adjacent to *HLA-DQA1* and *HLA-DRB1 *(Fig. 1B)*.*

To investigate subtype-specific effects, each JIA subtype was compared separately against the control group. Table 1 shows the twelve top SNPs that reached genome-wide significance in the association analysis, their chromosome position, the minor alleles and their frequencies in all JIA patients as one consolidated group. Top associated SNPs for each clinical JIA subtype group compared with the control group are shown in *Supplementary Table *2. Nine SNPs reached genome-wide significance level for the oligoarthritis subtype. Two SNPs on chromosome 6 (rs28421666 & rs2071550) reached genome-wide significance for the RF-negative subtype, and these were the same as the top SNPs for the oligoarthritis subtype.

**Table 1:** Results of association tests between JIA (*n* = 329) and controls (*n* = 748). Chr: Chromosome, MA: Minor allele, MAF: Minor allele frequency

				**Controls**	**All JIA (*n* = 329)**				
				**(*n*= 748)**					
**Chr**	**Lead SNP**	**Position**	**MA**	**MAF**	**MAF**	**P**	**FDR**	**OR**	**95% CI**
6	rs28421666	32592737	G	0.05	0.15	1.33E-12	7.81E-07	3.51	2.48–4.96
6	rs9272105	32599999	G	0.45	0.3	2.09E-10	6.16E-05	0.52	0.42–0.63
6	rs9273012	32611641	G	0.26	0.41	8.62E-10	2.00E-04	1.93	1.56–2.37
6	rs9272219	32602269	A	0.26	0.41	1.28E-09	2.00E-04	1.91	1.55–2.35
6	rs17576984	32212985	A	0.11	0.21	2.12E-09	3.00E-04	2.27	1.74–2.96
6	rs2395148	32321554	A	0.04	0.11	2.04E-08	2.10E-03	2.9	2–4.2
6	rs9501173	32279902	A	0.06	0.14	2.84E-08	2.10E-03	2.5	1.81–3.46
6	rs3749967	32283844	G	0.06	0.14	2.84E-08	2.10E-03	2.5	1.81–3.46
6	rs9469099	32308908	A	0.04	0.11	4.84E-08	3.10E-03	2.8	1.94–4.06
6	rs13192471	32671103	G	0.18	0.28	5.22E-08	3.10E-03	1.93	1.52–2.44
6	rs3763313	32376471	C	0.2	0.3	8.74E-08	4.40E-03	1.91	1.51–2.42
6	rs35120848	32670495	A	0.18	0.28	8.85E-08	4.40E-03	1.91	1.51–2.42

Two SNPs on chromosome 7 (rs3926927 & rs10245392) reached genome-wide significance levels for the subtype of systemic JIA.

### Association between the MHC alleles and JIA

To identify HLA alleles conveying genome-wide association signals on chromosome 6, classical HLA alleles in the major histocompatibility complex (MHC) region were imputed from SNP data (Table 2A* & Supplementary Table 3*)*.* In the *HLA-DRB1* locus, the DRB1*08:01 allele showed the strongest association, conferring an increased risk of JIA in the entire group (OR = 3.3, 95% CI: 2.76–5.67). A similar association was found for HLA-DQA1*04:01 (OR = 3.22, 95% CI: 2.58–5.21) and HLA-DQB1*04:02 (OR = 3.19, 95% CI: 2.51–5.10). Additionally, the HLA-DRB1*11 allelic group was associated with an increased risk of JIA (OR = 2.17, 95% CI: 1.46–2.89). We also performed conditional analysis to control for independence of associations within the HLA region for JIA as a group (Table 2B). First, the association was conditioned on the most significant marker, HLA-DRB1*08, which removed the signal for HLA-DQA1*04 and HLA-DQB1*04. This step left the HLA-DRB1*11 allele as the top hit (FDR = 1.00E-04). At the next step, after conditioning both on HLA-DRB1*08 and HLA-DRB1*11, HLA-DPB1*02 was the top association, although not statistically significant.

**Table 2:** **A**. Top hits from association tests of classical HLA alleles between A. JIA (*n* = 329) vs controls (*n* = 748), **B**. Subsequent conditional analysis, AF: allele frequency

**A**							
	**Controls (***n***= 748)**	**All JIA (***n***= 329)**					
**HLA**	**AF**	**AF**	**OR**	**95% CI**	**P**		**FDR**
DRB1*08	0.05	0.14	3.16	2.51–5.02	7.92E-13		5.75E-10
DRB1*08:01	0.04	0.13	3.3	2.76–5.67	8.75E-14		1.40E-10
DQA1*04	0.05	0.14	3.22	2.58–5.21	5.28E-13		5.26E-10
DQA1*04:01	0.05	0.14	3.22	2.58–5.21	5.28E-13		5.26E-10
DQB1*04	0.05	0.13	3.19	2.51–5.1	1.88E-12		6.26E-10
DQB1*04:02	0.05	0.14	3.19	2.51–5.1	1.88E-12		6.26E-10
DRB1*11	0.06	0.13	2.17	1.46–2.89	4.37E-05		1.10E-03
**B**							
**HLA**	**OR**	**95% CI**	**P**	**FDR**		**Conditioned on**	
DRB1*08	3.16	2.51–5.02	7.92E-13	5.75E-10		Initial association	
DRB1*11	2.45	1.76–3.4	1.01E-07	1.00E-04		DRB1*08	
DPB1*02	1.54	1.2–2	9.00E-04	1.09E-01		DRB1*08 & DRB1*11	

In the subtype-specific association analysis (*Supplementary Table 4)*, an increased risk associated with HLA-DRB1*08:01 was largely attributable to the subtypes oligoarthritis (OR = 4.98, 95% CI: 3.22–7.70) and RF-negative polyarthritis (OR = 4.5, 95% CI: 2.62–7.72). HLA-DQA1*04:01 (OR = 4.67, 95% CI: 3.05–7.15) and HLA-DQB1*04:02 (OR = 4.58, 95% CI: 2.99–7.02) were also significantly associated with increased disease risk of oligoarthritis. These two alleles were more frequent in the subgroup of RF-negative polyarthritis patients, with OR = 4.16, 95% CI: 2.45–7.09 and OR = 3.95, 95% CI: 2.31–6.75, respectively. The HLA-DRB1*11 allelic group was also associated with the oligoarthritis (OR = 2.61, 95% CI: 1.73–3.95). The frequencies of these alleles were not statistically significantly increased in any other subtypes of JIA. HLA-B*27:05 was the only allelic subgroup significantly associated with an increased risk of the ERA subtype (OR = 9.92, 95% CI: 4.27–23.04). Significant associations were not found for other subtypes, likely due to the low number of observations in these groups.

On the other hand, DRB1*07, DRB1*04 and DQA1*02, DQA1*03, DQB1*02, DQB1*03 and C*06 alleles were associated with reduced risk in oligoarthritis patients (FDR < 0.05), and DRB1*04, DQA1*03, DQB1*03 alleles were associated with reduced risk in RF-negative polyarthritis patients (FDR < 0.05).

### Association of classical HLA alleles with autoantibody status and disease phenotypes

To identify whether the defined HLA alleles are associated with disease phenotypes, information for sex (females), age at disease onset, autoantibody positivity for ANA, anti-CCP2, RF and disease subtypes were retrieved from medical charts and coded as binary variables. Phi coefficient ranging from -1 to 1 and related *P* values were calculated for JIA as a group *(Supplementary Fig. 2).* For the JIA group there was a strong significant positive correlation between HLA-DRB1*08 and HLA-DQA1*04 (phi = 0.97, *P* < 0.0001), HLA-DQB1*04 (phi = 0.95, *P* < 0.0001). These alleles correlated positively with ANA (phi = 0.22, *P* < 0.0001). HLA-DQA1*04 also had a significant positive correlation with HLA-DQB1*04 (phi = 0.98, *P* < 0.0001) and these alleles correlated positively with female sex (phi = 0.14–0.15, *P* < 0.001) and ANA (phi = 0.20–0.22, *P* < 0.001) but did not correlate with anti-CCP2 or RF. HLA-DRB1*11 only correlated with ANA (phi = 0.17, *P* = 0.004). ANA correlated positively with female sex (phi = 0.23, *P* < 0.0001) but did not correlate with anti-CCP2 positivity or with RF positivity. Anti-CCP2 and RF were positively correlated (phi = 0.64, *P* < 0.0001).

To further investigate associations of the defined haplotypes with disease phenotypes, patients carrying these alleles were compared to those who did not (Table 3). In this analysis, due to the high correlation between HLA-DRB1*08 and the two DQ alleles (HLA-DQA1*04 and HLA-DQB1*04) and because HLA-DRB1*08 allele was more frequent among patients, only this allele was considered. Compared to the HLA-DRB1*08-negative patients, the HLA-DRB1*08-positive group had significantly lower age at onset (6.7 vs 3.6 years, *P* = 0.005), higher frequency of females (80.2% vs 65.5%, *P* = 0.05) and frequency of ANA-positive patients (62.6% vs 36.5%, *P* = 0.001). All RF-positive polyarthritis patients within the cohort were HLA-DRB1*08-negative (*P* = 0.023). Positive patients for HLA-DRB1*11 exhibited a higher frequency of ANA positivity compared to HLA-DRB1*11-negaitive patients (57% vs. 39.6% *P* = 0.02). Only one single RF-positive polyarthritis patient and one ERA patient were positive for HLA-DRB1*11 alleles (the difference was only statistically significant in ERA subtype, *P* = 0.02). Age at onset and female ratio was not significantly different between HLA-DRB1*11 positive and negative group.

**Table 3:** Association of HLA-DRB1*08 and HLA-DRB1*11 with disease parameters in JIA

	DRB1*08 +	DRB1*08-	***OR***	***95% CI***	**Adjusted P**	DRB1*11 +	DRB1*11-	***OR***	***95% CI***	**Adjusted P**
	***n* = *91 (%)***	***n* = *238 (%)***				***n* = *79 (%)***	***n* = *250 (%)***			
Age at onset, median months (years) *n* = 300	43 (3.6)	81 (6.7)	NA	NA	0.005	53 (4.4)	81 (6.75)	NA	NA	1
Females *n* = 329	73 (80.2)	156 (65.5)	2.1	1.2–3.9	0.05	57 (72.1)	172 (68.9)	1.17	0.68–2.1	1
ANA-positive	57 (62.6)	87 (36.5)	2.69	1.6–4.5	0.001	45 (57)	99 (39.6)	2.22	1.3–3.8	0.02
RF-positive	2 (2.2)	14 (5.9)	0.35	0.078–1.41	1	0 (0)	16 (6.4)	0	0–0.83	0.13
Anti-CCP2-positive	3 (3.3)	15 (6.3)	0.47	0.14–1.6	1	1 (1.3)	17 (6.8)	0.17	0.016–1.05	0.4
Oligoarthritis *n* = 161	52 (57.1)	106 (44.5)	1.6	0.97–2.54	0.084	44 (55.7)	113 (45.2)	1.5	0.92–2.56	0.121
RF negative polyarthritis *n* = 87	26 (28.6)	63 (26.47)	1.23	0.7–2.1	0.491	22 (27.8)	69 (27.6)	1.01	0.57–1.75	0.999
Systemic *n* = 24	3 (3.3)	20 (8.4)	0.37	0.11–1.15	0.146	8 (10.1)	15 (6.0)	1.76	0.74–4.27	0.212
Enthesitis-related *n* = 22	5 (5.5)	18 (7.56)	0.71	0.3–1.9	0.633	1 (1.2)	22 (8.8)	0.13	0.01–0.74	0.021
Psoriatic *n* = 19	4 (4.4)	15 (6.3)	0.5	0.15–1.7	0.417	3 (3.8)	15 (6.0)	0.61	0.18–2.1	0.6
RF-positive polyarthritis *n* = 12	0 (0)	12 (5)	0	0–0.74	0.023	1 (1.26)	11(4.4)	0.25	0.023–1.14	0.25
Undifferentiated *n* = 4	1 (1.1)	3 (1.26)	0.86	0.06–5.8	0.99	0 (0)	4 (1.6)	0	0–3.2	0.575

Mann–Whitney test was utilized for age comparison, while Fisher's exact test was employed for analyzing all other parameters. *P*-values were adjusted for multiple comparisons using the Bonferroni method. ANA data was available for 297 patients, RF data for 197 patients, and CCP2 data for 300 patients

*NA* Not Applicable

### Association analyses of JIA defined by ANA status

Next, patients were stratified based on ANA status (positive vs negative), and association analysis for these groups was performed compared to healthy controls. We observed that the ANA-positive group associated significantly with DRB1*08, DQA1*04 and DQB1*04, previously found for JIA as one group compared to the control group, but at a higher significance level (Table 4A*, Supplementary Table 5 & Supplementary Fig. 3A*). In contrast, the ANA-negative group did not show significant associations with any of the classical HLA alleles (*Supplementary Fig. 3E*). In addition, stepwise logistic regression conditional analysis identified DRB1*11 allele as a separate signal (FDR = 2.60E-06) (Table 4B* & Supplementary Fig. 3B-D*) in the region. Further conditioning for both DRB1*08 and DRB1*11 left DPB1*02 allele as an independent association hit (FDR = 7.00E-03). On the other hand, DQB1*06, DRB1*15, DRB1*07, DRB1*04, DQA1*03, DQB1*02, C*06 and A*03 alleles were observed less frequently in ANA-positive patients (FDR < 0.05) suggesting that they are protective (*Supplementary Table 5*).

**Table 4:** **A**. Results of association tests of classical HLA alleles between ANA-positive JIA (*n* = 144) and controls (*n* = 748), and **B**. subsequent conditional analyses. AF: allele frequency

**A**										
***HLA***	**AF**			**AF**	**OR**		**95% CI**		**P**	**FDR**
	**JIA**			**Controls**						
DRB1*08	0.21			0.05	6.42		4.2–9.9		1.19E-17	8.97E-15
DRB1*08:01	0.19			0.04	7.03		4.6–11		4.00E-18	6.65E-15
DQA1*04	0.2			0.05	6.63		4.4–10.3		9.44E-18	8.97E-15
DQA1*04:01	0.2			0.05	6.63		4.4–10.3		9.44E-18	8.97E-15
DQB1*04	0.19			0.05	6.34		4.2–9.8		8.25E-17	2.86E-14
DQB1*04:02	0.19			0.05	6.34		4.2–9.8		8.25E-17	2.86E-14
DRB1*11	0.16			0.06	2.84		1.9–4.4		1.59E-06	6.61E-05
DRB1*11:01	0.1			0.04	2.83		1.8–4.7		3.57E-05	7.35E-04
DPB1*02	0.27			0.15	2.15		1.6–3		4.29E-06	1.29E-04
DPB1*02:01	0.27			0.15	2.19		1.6–3.1		2.35E-06	9.01E-05
DQB1*06	0.24			0.3	0.4		0.3–0.7		2.78E-04	3.62E-03
DQB1*06:02	0.07			0.16	0.4		0.3–0.7		2.78E-04	3.62E-03
B*27	0.12			0.07	2.22		1.5–3.5		3.43E-04	4.17E-03
B*27:05	0.11			0.06	2.22		1.5–3.5		4.83E-04	5.07E-03
A*02	0.46			0.37	1.62		1.3–2.2		4.31E-04	4.74E-03
A*02:01	0.45			0.36	1.63		1.3–2.2		3.50E-04	4.20E-03
DRB1*15	0.07			0.16	0.45		0.3–0.8		5.67E-04	5.77E-03
**B**										
**HLA**	**OR**			**95% CI**	**P**			**FDR**	**Conditioned on**	
DRB1*08	6.42			4.2–9.9	1.19E-17			8.97E-15	Initial association	
DRB1*11	3.68			2.5–5.7	1.88E-09			2.60E-06	DRB1*08	
DPB1*02	2.3			1.7–3.3	1.69E-06			7.00E-03	DRB1*08, DRB1*11	
B*51	0.31			0.2–0.8	6.51E-03			2.80E-01	DRB1*08, DRB1*11, DPB1*02	

We performed additional comparisons between the ANA-positive and ANA-negative JIA groups in our study (Table 5). It was observed that ANA-positive patients had a significantly higher percentage of DRB1*08, 39.6% vs 19.6% in the ANA-negative group (*P* = 0.0016). The DQA1*04 frequency was 38.2% in the ANA-positive group and 18.3% in the ANA-negative group (*P* = 0.0016), DQB1*04 frequencies were 36.11% in ANA-positive vs 18.3% in ANA-negative patients (*P* = 0.0016) and DRB1*11 frequencies were 31.2% in ANA-positive and 17% in ANA-negative patients (*P* = 0.032). The ANA-positive group also had a higher frequency of females (80.5% vs 59.4%) than the ANA-negative group (*P* = 0.0008), and age at disease onset was significantly lower in the ANA-positive group, 3 vs 8 years in the ANA-negative group (*P* = 0.0016).

**Table 5:** Association of ANA with predisposing alleles in JIA (*n* = 297) and disease parameters phenotypes

	**ANA-positive (***n***= 144) (%)**	**ANA-negative (***n***= 153) (%)**	**Adjusted P**
DRB1*08	57 (39.6)	30 (19.6)	0.0016
DQA1*04	55 (38.2)	28 (18.3)	0.0016
DQB1*04	52 (36.1)	28 (18.3)	0.0016
DRB1*11	45 (31.2)	26 (17)	0.032
Sex-females	116 (80.5)	91 (59.4)	0.0008
Age at onset median months (years)	37 (3)	99 (8.2)	0.0016
RF-positive	7 (4.9)	9 (5.9)	1
Anti-CCP2-positive	8 (5.5)	10 (6.5)	1
Oligoarthritis *n* = 151	88 (61.1)	63 (41.2)	0.0007
RF-negative polyarthritis *n* = 75	37 (25.7)	38 (24.8)	0.9
Systemic *n* = 18	3 (2.0)	15 (9.8)	0.006
Enthesitis-related *n* = 20	4 (2.8)	16 (10.4)	0.01
Psoriatic *n* = 17	4 (2.8)	13 (8.5)	0.04
RF-positive polyarthritis *n* = 12	7 (4.9)	5 (3.3)	0.56
Undifferentiated *n* = 4	1 (0.7)	3 (2)	0.62

Mann–Whitney test was employed for the comparison of Age, while Fisher's exact test was utilized for the analysis of all other parameters. *P*-values were adjusted for multiple comparisons using the Bonferroni method. RF data was available for 197 patients, and CCP2 data was available for 300 patients

Among the 297 patients with available ANA data, 144 (48%) tested positive for ANA. While the majority of ANA-positive patients were of the oligoarthritis (88 patients, 61%) and RF-negative polyarthritis (37 patients, 25%) subtypes, statistical significance was observed only within the oligoarthritis group. Additionally, 35 patients (13%) of ANA-positive JIA were distributed across other subtypes. sJIA, ERA and psoriatic patients were significantly more common in ANA-negative group.

Furthermore, additional analysis was undertaken to mitigate potential bias toward oligoarthritis patients within the ANA-positive subgroup. We conducted a comparison of ANA-positive and ANA-negative patients regarding the positivity for DRB1*08, considering both oligoarthritis and RF-negative polyarthritis subtypes as one group. By considering both oligoarthritis and RF- polyarthritis patients within a unified group, we aimed to capture the shared genetic background between these subtypes, as supported by existing literature [20–22].

Our observations revealed that among DRB1*08-positive patients, 70.7% were ANA-positive, while 29.3% were ANA-negative (OR = 2.64, 95% CI: 1.4–4.7) (Table 6). Similar results were observed when restricting the analysis to oligoarticular JIA patients alone (data not included).

**Table 6:** Association of the HLA-DRB1*08 with Oligo & RF-negative polyarthritis patients in JIA

	DRB1*08 +	DRB1*08-	***OR***	***95% CI***	***Adjusted P***
	***n* = *75 (%)***	***n* = *151 (%)***			
ANA-positive Oligo & RF-negative polyarthritis *n* = 125	53 (70.7)	72 (47.7)	2.64	1.4–4.7	0.001
ANA-negative Oligo & RF-negative polyarthritis *n* = 101	22 (29.3)	79 (52.3)			

Finally, we employed logistic regression analysis to evaluate the association between found significant variables, namely DRB1*08, DRB1*11, sex and age at onset, in relation to ANA status. Considering ANA status as the dependent variable, logistic regression with independent variables, DRB1*08, DRB1*11, sex, and age at onset demonstrated high statistical significance (*Supplementary Table 6*). The present analysis revealed that DRB1*08 and DRB1*11, sex and age at onset were in the equation, implying that they were all contributing to the risk of ANA positivity in JIA patients with different effect sizes. Of these, DRB1*08, DRB1*11, and female sex showed the highest statistically significant B regression coefficients: 0.79, 0.77, and 0.75, respectively, while age at onset had a smaller yet statistically significant coefficient of -0.01, suggesting their roles in promoting an ANA-positive profile in JIA.

## Discussion

The genome-wide association analysis revealed significant associations between JIA and multiple single nucleotide polymorphisms (SNPs) located on chromosome 6 within the MHC class II gene region. Notably, the top SNP, rs28421666, was adjacent to *HLA-DQA1* and *HLA-DRB1*. These findings are consistent with previous reports suggesting that the HLA region plays a crucial role in JIA susceptibility. In our study, we found an association of specific alleles (HLA-DRB1*08, HLA-DQA1*04:01, HLA-DQB1*04:02, and HLA-DRB1*11) with increased risk of JIA, which supports the idea that certain HLA alleles confer susceptibility to the disease.

An extended *HLA-DRB1-DQA1-DQB1* haplotype has consistently been implicated as conferring increased risk to JIA [8, 23–28]. It has been known that strong linkage disequilibrium (LD) exists between DRB1*08, DQA1*04, and DQB1*04 alleles, and thus it becomes challenging to ascertain which of these HLA class II genes are primarily involved in JIA [29]. According to our analysis of the frequency of these alleles among patients and controls, four patients carried DRB1*08 without DQA1*04:01 and DQB1*04:02, while seven patients carried DRB1*08 but not DQB1*04:02. Among controls, four individuals carried DRB1*08 without DQA1*04 and three had DRB1*08 but did not carry DQB1*04. These findings imply that within this haplotype, the DRB1*08 allele could be the main allele linked to increased vulnerability to developing JIA. Meanwhile, the connection with DQA1*04 and DQB1*04 might be due to their linkage to DRB1*08 through LD. This observation is in line with results from Norwegian and Polish cohorts where they concluded that in *DRB1-DQA1-DQB1* haplotype, DRB1*08 allele is primarily associated with pauciarticular and RF-negative polyarticular JIA [29].

In accordance with previous studies, our study found strong subtype-specific effects for HLA-DRB1*08, HLA-DQA1*04:01, HLA-DQB1*04:02 alleles, particularly for oligoarthritis and RF-negative polyarthritis, which suggests similar genetic predisposition for these two subtypes [8, 23–28]. Although these alleles did not reach a statistical significance level in other subtypes, they were elevated in some; 22% of ERA and 21% of psoriatic patients as compared to 10% of healthy controls, had HLA-DRB1*08.

We did not find any other distinct statistically significant HLA associations for other subtypes except for ERA patients, which had a significant association with HLA-B*27. The small number of patients in other subtypes might explain this nonsignificant association, but it also may reflect heterogeneity and different genetic backgrounds for these subtypes. This subtype-specific genetic architecture aligns with the heterogeneous nature of JIA and underscores the importance of considering distinct subtypes when investigating genetic risk factors.

The relationship between HLA genetic variants and autoantibodies is a critical aspect of autoimmune diseases. In this study, ANA positivity emerged as a significant marker associated with specific HLA alleles: DRB1*08, DRB1*11 and DPB1*02. This observation suggests that specific HLA alleles might predispose individuals to develop JIA with ANA positivity. The observed correlations between specific HLA alleles and ANA positivity, along with associations with female sex and younger age at onset, suggest potential interactions between genetic factors and autoantibody production in JIA.

One peak incidence of JIA is known to occur during early childhood, typically between the ages of 1 and 4 years, with most of these patients presenting with an oligoarticular phenotype and commonly testing positive for ANAs. ANA-positive JIA patients are also at a higher risk of chronic anterior uveitis, distinct from the acute anterior uveitis observed in both adult and paediatric spondyloarthritis (SpA), which has no counterpart in adult-onset arthritis. This underscores the unique clinical presentation and disease progression in pediatric patients, which necessitates a tailored approach to management and treatment.

In a previous study, it was reported that among polyarticular JIA patients, there was a significant correlation between earlier age of onset and the presence of DRB1*08 or DRB1*11 alleles: 4 years compared to 8 years for those who do not have the predisposing allele. They observed the same age effect trend in oligoarthritis patients; however, it was not statistically significant. They also noted an independent association for DPB1*02:01 allele with earlier age of onset [23]. In our study, we observed a consistent age-related effect associated with DRB1*08. Patients carrying DRB1*08 showed an average age at onset of 3.6 years, whereas those without the allele had an onset of 6.7 years. Additionally, the comparison of ANA-positive and ANA-negative JIA patients unveiled significant differences in terms of HLA alleles, sex, and age at onset: 61% of ANA positive patients were oligoarthritis, followed by 26% being RF-negative polyarthritis. The fact that ANA-positive patients were more frequently oligoarthritis, DRB1*08 or DRB1*11 positive, female and had a younger age at onset (3 years vs 8 years) than ANA-negative patients further support the notion that ANA positivity is a genetically driven phenomenon associated with a specific subgroup of JIA patients. Furthermore, the logistic regression analysis reaffirmed the effects of HLA alleles, female sex, and lower age at onset on ANA positivity, with DRB1*08, DRB1*11, and female sex having a larger effect than age at onset, suggesting that these factors collectively contribute to the complex autoimmunity in ANA positive JIA. Interestingly, although seven out of twelve (~ 60%) RF-positive polyarthritis patients had ANA, none of them were positive for DRB1*08 and only one had DRB1*11. This suggests a distinct genetic predisposition for this subtype, independent of their ANA status. The findings also raise the question of whether ANA positivity could serve as a marker to stratify JIA subtypes. The higher prevalence of ANA positivity in certain subtypes, such as oligoarthritis, and its genetic association with specific HLA alleles, suggests ANA status could potentially help refine JIA subtyping and treatment strategies. However, further research is needed to determine the clinical utility of using ANA positivity as a stratification factor.

These findings are in accordance with the classification introduced by the Paediatric Rheumatology International Trials Organization (PRINTO), which identifies a category of arthritis termed 'early-onset ANA-positive JIA' [30]. They suggested that these patients form a distinct homogeneous subgroup, currently classified in different subtypes, irrespective of the course of joint disease [22, 31, 32]. This proposal, if validated by further research, could lead to a more nuanced classification of JIA [20]. Our findings of the specific associations between HLA-DR and ANA status in JIA are consistent with the notion proposed in this review for a new classification of JIA, incorporating more molecular biological phenotyping for disease subgroups.

So far, the strongest genetic association for systemic JIA has been with *HLA-DRB1-11* [33] but no HLA associations were found in our study. We identified two SNPs on chromosome 7 that reached genome-wide significance levels for the systemic JIA subtype in our cohort. Both are located adjacent to the *RNU6* (U6 Small Nuclear RNA) gene, and with no prior reports in the literature to our knowledge. Given the small size of this sJIA cohort, it is imperative to conduct further investigation and validation in larger cohorts to confirm the existence of a genuine association before drawing definitive conclusions.

Our study's strengths include its use of a well-defined cohort of Swedish JIA patients, diagnosed according to the ILAR criteria, as well as the incorporation of genetic, antibody and clinical patient data. However, some limitations should be considered. First, the cohort size, while valuable given the rarity of the disease, may limit the detection of associations with smaller effect sizes and in the smaller subtypes. Additionally, this investigation is performed on a Swedish cohort, and therefore may not fully generalize to other populations with different genetic backgrounds. Another limitation is the retrospective nature of data collection which is subject to missing and possibly inaccurate data specially in the absence of mutual criteria for ANA positivity. Moreover, the lack of available clinical information and autoantibodies in some patients may have affected the analysis and results. Similarly, there was an age discrepancy between the control cohort and the JIA cohort. This age difference might potentially impact allele frequencies across different generations, which could have influenced our study results. On the other hand, including additional clinical data—such as ANA specificities, patients' uveitis status and its progression, and comprehensive follow-up data on JIA disease progression—could have enhanced the depth of our study.

## Conclusions

The results of our study highlight the potential role of specific HLA alleles in the development of ANA in JIA patients. These findings could have important implications for understanding the underlying genetic factors contributing to the pathogenesis of JIA, and they have the potential to refine classifications and improve therapeutic approaches for JIA patients in the future.

### Supplementary Information

Supplementary Material 1.

## Data Availability

According to Swedish law individuals data is not publicly available but the datasets generated and/or analysed during the current study are available on reasonable request.

## Abbreviations

ACPA: Anti Cyclic Citrullinated Peptide Antibodies
ANA: Anti Nuclear Antibodies
CCP: Cyclic Citrullinated Peptides
ERA: Enthesitis Related Arthritis
EIMS: Epidemiological Investigation of Multiple Sclerosis
ELISA: Enzyme Linked Immunosorbent Assay
FDR: False Discovery Rate
FEIA: Fluorescence Enzyme Immunoassay
GWAS: Genome-wideAssociation Study
HLA: Human Leukocyte Antigen
IgG: Immunoglobulin G
ILAR: International League of Associations for Rheumatology
JABBA: Juvenile Arthritis BioBank
JIA: Juvenile Idiopathic Arthritis
LD: Linkage Disequilibrium
MAF: Minor Allele Frequency
MHC: Major Histocompatibility Complex
PCA: Principal Component Analysis
PRINTO: Paediatric Rheumatology International Trials Organization
QQ: Quantile–Quantile
RA: Rheumatoid Arthritis
RF: Rheumatoid Factor
SNP: Single Nucleotide Polymorphism
SNP2HLA: Single Nucleotide Polymorphism to HLA
T1DGC: Type 1 Diabetes Genetics Consortium
λGC: Genomic Control Inflation Factor
