# Supplementary material for: Genetic association of antinuclear antibodies with HLA in JIA patients: a Swedish cohort study
Source: Pediatr Rheumatol Online J. 2024 Aug 26;22:79. doi: 10.1186/s12969-024-01017-8 (PMC11348572; doi:10.1186/s12969-024-01017-8)
Supplement: Supplementary file 1 — Supplementary Material 1. [file 12969_2024_1017_MOESM1_ESM.docx]

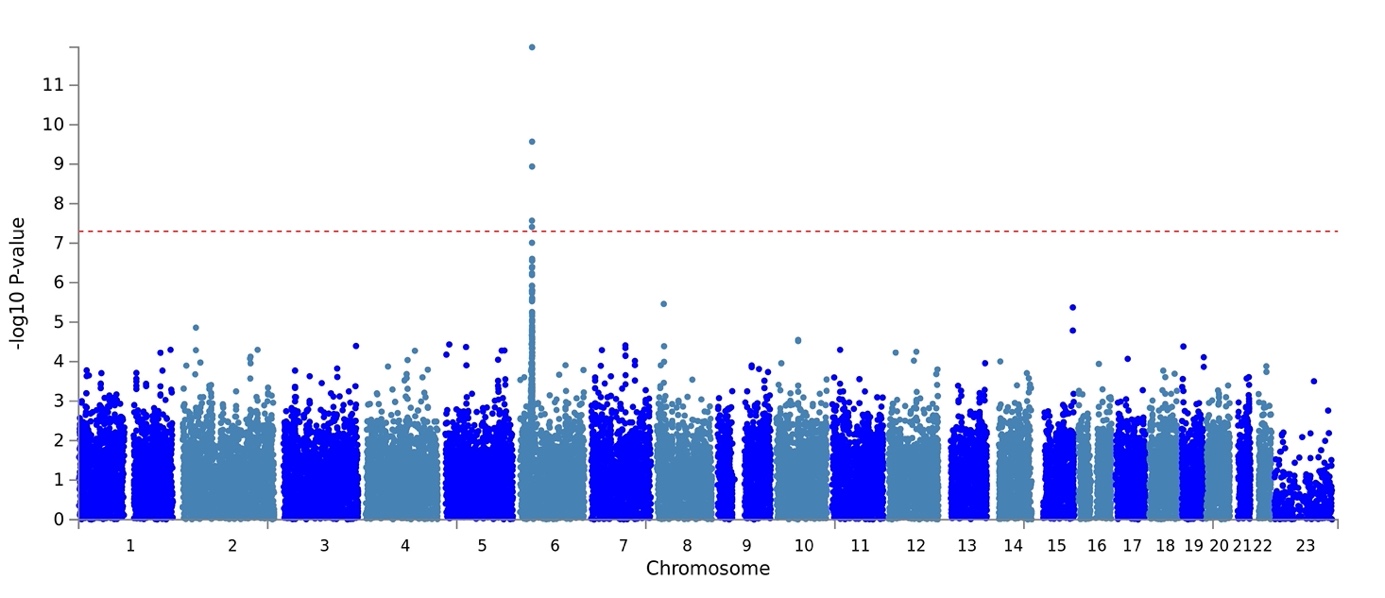


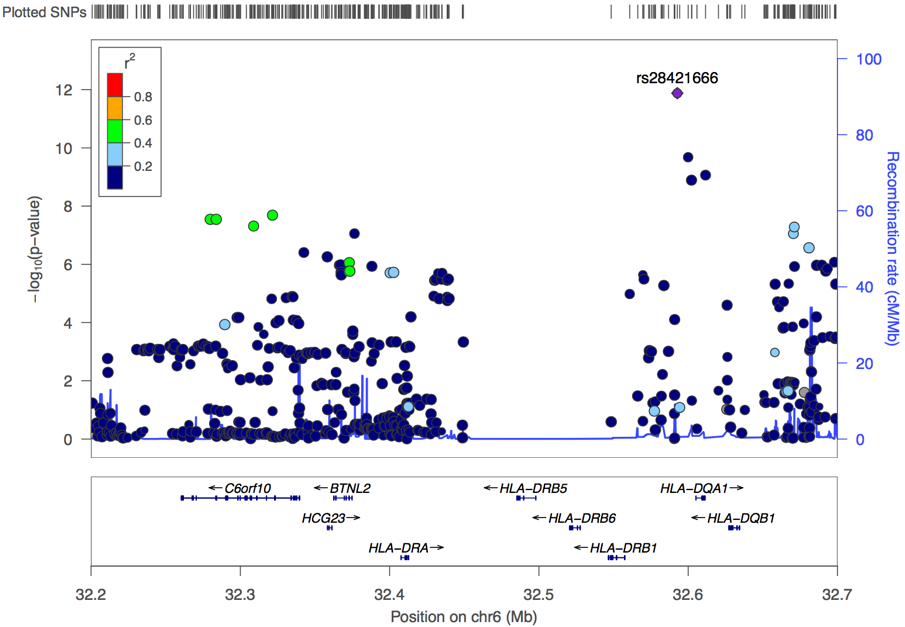


**A.**  **B.**

**Figure 1**. **A**. Manhattan plot of the genome-wide association of 329 JIA patients compared to 748 controls. The −log_10_ P values for each SNP in the association tests are shown on the y axis and the chromosomes are ordered on the x axis. Twelve genetic loci in the HLA region surpassed the genome-wide significance threshold (P<5x10^-8^= −log10(P) > 7.3; indicated by the red dotted line). **B.** Regional association plot of the genome‐wide association of JIA with SNPs at chromosome 6 (Chr6). The top associated SNP (rs28421666) at the HLA locus is located in between *HLA-DRB1* and *HLA-DQA1.* The level of linkage disequilibrium between SNPs in the zoomed in regions of chromosome 6 is indicated by r^2^.

**Table 1.** Results of association tests between JIA (n=329) and controls (n=748). Chr: Chromosome, MA: Minor allele, MAF: Minor allele frequency

|  |  |  |  |  | **Controls** |  | **All JIA (n=329)** | | | |  |
| --- | --- | --- | --- | --- | --- | --- | --- | --- | --- | --- | --- |
|  |  |  |  |  | **(n=748)** |  |  |  |  |  |  |
| **Chr** | **Lead SNP** | **Position** | **MA** |  | **MAF** |  | **MAF** | **P** | **FDR** | **OR** | **95% CI** |
| 6 | rs28421666 | 32592737 | G |  | 0.05 |  | 0.15 | 1.33E-12 | 7.81E-07 | 3.51 | 2.48-4.96 |
| 6 | rs9272105 | 32599999 | G |  | 0.45 |  | 0.3 | 2.09E-10 | 6.16E-05 | 0.52 | 0.42-0.63 |
| 6 | rs9273012 | 32611641 | G |  | 0.26 |  | 0.41 | 8.62E-10 | 2.00E-04 | 1.93 | 1.56-2.37 |
| 6 | rs9272219 | 32602269 | A |  | 0.26 |  | 0.41 | 1.28E-09 | 2.00E-04 | 1.91 | 1.55-2.35 |
| 6 | rs17576984 | 32212985 | A |  | 0.11 |  | 0.21 | 2.12E-09 | 3.00E-04 | 2.27 | 1.74-2.96 |
| 6 | rs2395148 | 32321554 | A |  | 0.04 |  | 0.11 | 2.04E-08 | 2.10E-03 | 2.9 | 2-4.2 |
| 6 | rs9501173 | 32279902 | A |  | 0.06 |  | 0.14 | 2.84E-08 | 2.10E-03 | 2.5 | 1.81-3.46 |
| 6 | rs3749967 | 32283844 | G |  | 0.06 |  | 0.14 | 2.84E-08 | 2.10E-03 | 2.5 | 1.81-3.46 |
| 6 | rs9469099 | 32308908 | A |  | 0.04 |  | 0.11 | 4.84E-08 | 3.10E-03 | 2.8 | 1.94-4.06 |
| 6 | rs13192471 | 32671103 | G |  | 0.18 |  | 0.28 | 5.22E-08 | 3.10E-03 | 1.93 | 1.52-2.44 |
| 6 | rs3763313 | 32376471 | C |  | 0.2 |  | 0.3 | 8.74E-08 | 4.40E-03 | 1.91 | 1.51-2.42 |
| 6 | rs35120848 | 32670495 | A |  | 0.18 |  | 0.28 | 8.85E-08 | 4.40E-03 | 1.91 | 1.51-2.42 |

**Table 2 A.** Top hits from association tests of classical HLA alleles between A. JIA (n=329) vs controls (n=748), **B.** Subsequent conditional analysis, AF: allele frequency

|  |  | **Controls** |  | **All JIA (n=329)** | | | | |
| --- | --- | --- | --- | --- | --- | --- | --- | --- |
|  |  | **(n=748)** |  |  |  |  |  |  |
| **HLA** |  | **AF** |  | **AF** | **OR** | **95% CI** | **P** | **FDR** |
| *DRB1*08* |  | 0.05 |  | 0.14 | 3.16 | 2.51-5.02 | 7.92E-13 | 5.75E-10 |
| *DRB1*08:01* |  | 0.04 |  | 0.13 | 3.3 | 2.76-5.67 | 8.75E-14 | 1.40E-10 |
| *DQA1*04* |  | 0.05 |  | 0.14 | 3.22 | 2.58-5.21 | 5.28E-13 | 5.26E-10 |
| *DQA1*04:01* |  | 0.05 |  | 0.14 | 3.22 | 2.58-5.21 | 5.28E-13 | 5.26E-10 |
| *DQB1*04* |  | 0.05 |  | 0.13 | 3.19 | 2.51-5.1 | 1.88E-12 | 6.26E-10 |
| *DQB1*04:02* |  | 0.05 |  | 0.14 | 3.19 | 2.51-5.1 | 1.88E-12 | 6.26E-10 |
| *DRB1*11* |  | 0.06 |  | 0.13 | 2.17 | 1.46-2.89 | 4.37E-05 | 1.10E-03 |

**A.**

| **HLA** | **OR** | **95% CI** | **P** | **FDR** | **Conditioned on** |
| --- | --- | --- | --- | --- | --- |
| *DRB1*08* | 3.16 | 2.51-5.02 | 7.92E-13 | 5.75E-10 | Initial association |
| *DRB1*11* | 2.45 | 1.76-3.4 | 1.01E-07 | 1.00E-04 | *DRB1*08* |
| *DPB1*02* | 1.54 | 1.2-2 | 9.00E-04 | 1.09E-01 | *DRB1*08 & DRB1*11* |

**B.**

**Table 3**. Association of *HLA-DRB1*08* and *HLA-DRB1*11* with disease parameters in JIA.

|  | ***DRB1*08+*** | ***DRB1*08-*** | ***OR*** | ***95% CI*** | **adjusted P** | ***DRB1*11+*** | ***DRB1*11-*** | ***OR*** | ***95% CI*** | **Adjusted P** |
| --- | --- | --- | --- | --- | --- | --- | --- | --- | --- | --- |
|  | ***n= 91 (%)*** | ***n=238 (%)*** |  |  |  | ***n=79 (%)*** | ***n=250 (%)*** |  |  |  |
| Age at onset, median months (years) n=300 | 43 (3.6) | 81 (6.7) | NA | NA | 0.005 | 53 (4.4) | 81 (6.75) | NA | NA | 1 |
| Females n= 329 | 73 (80.2) | 156 (65.5) | 2.1 | 1.2-3.9 | 0.05 | 57 (72.1) | 172 (68.9) | 1.17 | 0.68-2.1 | 1 |
| ANA-positive | 57 (62.6) | 87 (36.5) | 2.69 | 1.6-4.5 | 0.001 | 45 (57) | 99 (39.6) | 2.22 | 1.3-3.8 | 0.02 |
| RF-positive | 2 (2.2) | 14 (5.9) | 0.35 | 0.078-1.41 | 1 | 0 (0) | 16 (6.4) | 0 | 0-0.83 | 0.13 |
| Anti-CCP2-positive | 3 (3.3) | 15 (6.3) | 0.47 | 0.14-1.6 | 1 | 1 (1.3) | 17 (6.8) | 0.17 | 0.016-1.05 | 0.4 |
| Oligoarthritis n=161 | 52 (57.1) | 106 (44.5) | 1.6 | 0.97-2.54 | 0.084 | 44 (55.7) | 113 (45.2) | 1.5 | 0.92-2.56 | 0.121 |
| RF negative polyarthritis n=87 | 26 (28.6) | 63 (26.47) | 1.23 | 0.7-2.1 | 0.491 | 22 (27.8) | 69 (27.6) | 1.01 | 0.57-1.75 | 0.999 |
| Systemic n=24 | 3 (3.3) | 20 (8.4) | 0.37 | 0.11-1.15 | 0.146 | 8 (10.1) | 15 (6.0) | 1.76 | 0.74-4.27 | 0.212 |
| Enthesitis-related n=22 | 5 (5.5) | 18 (7.56) | 0.71 | 0.3-1.9 | 0.633 | 1 (1.2) | 22 (8.8) | 0.13 | 0.01-0.74 | 0.021 |
| Psoriatic n=19 | 4 (4.4) | 15 (6.3) | 0.5 | 0.15-1.7 | 0.417 | 3 (3.8) | 15 (6.0) | 0.61 | 0.18-2.1 | 0.6 |
| RF-positive polyarthritis n=12 | 0 (0) | 12 (5) | 0 | 0-0.74 | 0.023 | 1 (1.26) | 11(4.4) | 0.25 | 0.023-1.14 | 0.25 |
| Undifferentiated n=4 | 1 (1.1) | 3 (1.26) | 0.86 | 0.06-5.8 | 0.99 | 0 (0) | 4 (1.6) | 0 | 0-3.2 | 0.575 |

Mann-Whitney test was utilized for age comparison, while Fisher's exact test was employed for analyzing all other parameters. P-values were adjusted for multiple comparisons using the Bonferroni method. ANA data was available for 297 patients, RF data for 197 patients, and CCP2 data for 300 patients. NA: Not Applicable.

**Table 4**. **A.** Results of association tests of classical HLA alleles between ANA-positive JIA (n=144) and controls (n=748), and **B.** subsequent conditional analyses. AF: allele frequency

| **HLA** | **AF** | **AF** | **OR** | **95% CI** | **P** | **FDR** |
| --- | --- | --- | --- | --- | --- | --- |
|  | **JIA** | **Controls** |  |  |  |  |
| *DRB1*08* | 0.21 | 0.05 | 6.42 | 4.2-9.9 | 1.19E-17 | 8.97E-15 |
| *DRB1*08:01* | 0.19 | 0.04 | 7.03 | 4.6-11 | 4.00E-18 | 6.65E-15 |
| *DQA1*04* | 0.2 | 0.05 | 6.63 | 4.4-10.3 | 9.44E-18 | 8.97E-15 |
| *DQA1*04:01* | 0.2 | 0.05 | 6.63 | 4.4-10.3 | 9.44E-18 | 8.97E-15 |
| *DQB1*04* | 0.19 | 0.05 | 6.34 | 4.2-9.8 | 8.25E-17 | 2.86E-14 |
| *DQB1*04:02* | 0.19 | 0.05 | 6.34 | 4.2-9.8 | 8.25E-17 | 2.86E-14 |
| *DRB1*11* | 0.16 | 0.06 | 2.84 | 1.9-4.4 | 1.59E-06 | 6.61E-05 |
| *DRB1*11:01* | 0.1 | 0.04 | 2.83 | 1.8-4.7 | 3.57E-05 | 7.35E-04 |
| *DPB1*02* | 0.27 | 0.15 | 2.15 | 1.6-3 | 4.29E-06 | 1.29E-04 |
| *DPB1*02:01* | 0.27 | 0.15 | 2.19 | 1.6-3.1 | 2.35E-06 | 9.01E-05 |
| *DQB1*06* | 0.24 | 0.3 | 0.4 | 0.3-0.7 | 2.78E-04 | 3.62E-03 |
| *DQB1*06:02* | 0.07 | 0.16 | 0.4 | 0.3-0.7 | 2.78E-04 | 3.62E-03 |
| *B*27* | 0.12 | 0.07 | 2.22 | 1.5-3.5 | 3.43E-04 | 4.17E-03 |
| *B*27:05* | 0.11 | 0.06 | 2.22 | 1.5-3.5 | 4.83E-04 | 5.07E-03 |
| *A*02* | 0.46 | 0.37 | 1.62 | 1.3-2.2 | 4.31E-04 | 4.74E-03 |
| *A*02:01* | 0.45 | 0.36 | 1.63 | 1.3-2.2 | 3.50E-04 | 4.20E-03 |
| *DRB1*15* | 0.07 | 0.16 | 0.45 | 0.3-0.8 | 5.67E-04 | 5.77E-03 |

**A.**

| **HLA** | **OR** | **95% CI** | **P** | **FDR** | **Conditioned on** |
| --- | --- | --- | --- | --- | --- |
| *DRB1*08* | 6.42 | 4.2-9.9 | 1.19E-17 | 8.97E-15 | Initial association |
| *DRB1*11* | 3.68 | 2.5-5.7 | 1.88E-09 | 2.60E-06 | *DRB1*08* |
| *DPB1*02* | 2.3 | 1.7-3.3 | 1.69E-06 | 7.00E-03 | *DRB1*08, DRB1*11* |
| *B*51* | 0.31 | 0.2-0.8 | 6.51E-03 | 2.80E-01 | *DRB1*08, DRB1*11, DPB1*02* |

**B.**

**Table 5.** Association of ANA with predisposing alleles in JIA (n=297) and disease parameters phenotypes

|  | **ANA-positive (n=144) (%)** | **ANA-negative (n=153) (%)** | **Adjusted P** |
| --- | --- | --- | --- |
| *DRB1*08* | 57 (39.6) | 30 (19.6) | 0.0016 |
| *DQA1*04* | 55 (38.2) | 28 (18.3) | 0.0016 |
| *DQB1*04* | 52 (36.1) | 28 (18.3) | 0.0016 |
| *DRB1*11* | 45 (31.2) | 26 (17) | 0.032 |
| Sex-females | 116 (80.5) | 91 (59.4) | 0.0008 |
| Age at onset median months (years) | 37 (3) | 99 (8.2) | 0.0016 |
| RF-positive | 7 (4.9) | 9 (5.9) | 1 |
| Anti-CCP2-positive | 8 (5.5) | 10 (6.5) | 1 |
| Oligoarthritis n=151 | 88 (61.1) | 63 (41.2) | 0.0007 |
| RF-negative polyarthritis n=75 | 37 (25.7) | 38 (24.8) | 0.9 |
| Systemic n=18 | 3 (2.0) | 15 (9.8) | 0.006 |
| Enthesitis-related n=20 | 4 (2.8) | 16 (10.4) | 0.01 |
| Psoriatic n=17 | 4 (2.8) | 13 (8.5) | 0.04 |
| RF-positive polyarthritis n=12 | 7 (4.9) | 5 (3.3) | 0.56 |
| Undifferentiated n=4 | 1 (0.7) | 3 (2) | 0.62 |

Mann-Whitney test was employed for the comparison of Age, while Fisher's exact test was utilized for the analysis of all other parameters. P-values were adjusted for multiple comparisons using the Bonferroni method. RF data was available for 197 patients, and CCP2 data was available for 300 patients.

**Table 6.** Association of the *HLA-DRB1*08* with Oligo & RF-negative polyarthritis patients in JIA.

|  | ***DRB1*08+*** | ***DRB1*08-*** | ***OR*** | ***95% CI*** | ***adjusted P*** |
| --- | --- | --- | --- | --- | --- |
|  | ***n= 75 (%)*** | ***n= 151 (%)*** |  |  |  |
| ANA-positive Oligo & RF-negative polyarthritis n= 125 | 53 (70.7) | 72 (47.7) | 2.64 | 1.4-4.7 | 0.001 |
| ANA-negative Oligo & RF-negative polyarthritis n= 101 | 22 (29.3) | 79 (52.3) |  |  |  |

**Supplementary Material:**


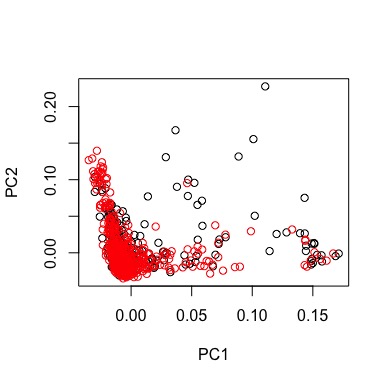


JIA patients

Controls

**
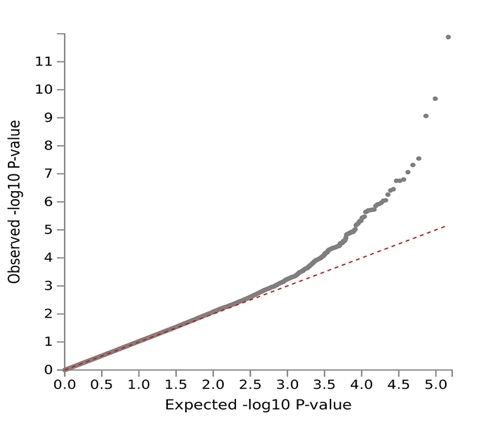
**

1. **B.**

**Supplementary Figure 1**. **A**. Principal Component Analysis (PCA) for Genome-Wide Association Study (GWAS). **B**. Quantile-Quantile plot displaying the expected (x-axis) and observed (y-axis) −log10(P value) distribution in GWAS analysis after correction for sex and the first 10 principal components. PCA was conducted on SNPs with a minor allele frequency ≥ 5% and pruned for linkage disequilibrium at an r2 threshold of 0.2. Inflation factor (Lambda) = 1.02.

**Supplementary Table 1**. Clinical and laboratory data for the total JIA cohort grouped by ILAR definitions and HLA typing frequencies obtained by imputation.

| **ILAR category** | **Number** | **Females** | **Age at onset** | **ANA+** | **Anti-CCP2+** | **RF+** | ***DRB1*08+*** | ***DQA1*04+*** | ***DQB1*04+*** | ***DRB1*11+*** |
| --- | --- | --- | --- | --- | --- | --- | --- | --- | --- | --- |
|  |  |  | **mean month** |  |  |  |  |  |  |  |
| All JIA | 329 | 229 (70) | 79.7 | 144/297 (48.5) | 18/300 (6) | 17/197 (8.6) | 91 (27.6) | 87 (26.4) | 84 (25.5) | 79 (24) |
| Oligoarthritis (%) | 161 (49) | 105 (65.2) | 70.5 | 88/151 (58.3) | 6/151 (4) | 2/93 (2.15) | 52 (32.3) | 50 (31) | 48 (29.8) | 45 (27.9) |
| RF-negative polyarthritis (%) | 87 (26.4) | 74 (85) | 68 | 37/75 (49.3) | 2/76 (2.6) | 0/52 (0) | 26 (29.9) | 25 (28.7) | 24 (27.5) | 21 (24.1) |
| Systemic (%) | 24 (7.3) | 15 (62.5) | 80.5 | 3/18 (16.7) | 0/20 (0) | 0/11 (0) | 3 (12.5) | 3 (12.5) | 3 (12.5) | 8 (33.3) |
| Enthesitis-related (%) | 22 (6.7) | 8 (36.4) | 136.7 | 4/20 (20) | 0/20 (0) | 0/15 (0) | 5 (22.7) | 5 (22.7) | 5 (22.7) | 1 (4.5) |
| Psoriatic (%) | 19 (5.8) | 11 (57.9) | 98.9 | 4/17 (23.5) | 0/17 (0) | 0/11 (09) | 4 (21) | 3 (15.8) | 3 (15.8) | 3 (15.8) |
| RF-positive polyarthritis (%) | 12 (3.6) | 12 (100) | 123.5 | 7/12 (58.3) | 8/12 (66.7) | 12/12 (100) | 0 (0) | 0 (0) | 0 (0) | 1 (8.3) |
| Undifferentiated (%) | 4 (1.2) | 4 (100) | 122 | 1/4 (25) | 2/4 (50) | 3/3 (100) | 1 (25) | 1 (25) | 1 (25) | 0 (0) |
| Healthy Controls (%) | 748 | 568 (75.9) | NA | NA | NA | NA | 74 (9.9) | 70 (9.35) | 71 (9.5) | 90 (12) |

**Supplementary Table 2.** Results of association tests between JIA ILAR subtypes and controls (n=748). Chr: Chromosome, MA: Minor allele, F.: Minor allele frequency

|  |  |  |  | **Oligoarthritis** **(n=157)** | | | | |  |  |  |  |  |  | **RF-negative polyarthritis (n=91)** | | | | | |
| --- | --- | --- | --- | --- | --- | --- | --- | --- | --- | --- | --- | --- | --- | --- | --- | --- | --- | --- | --- | --- |
| **Chr** | **SNP** | **Position** | **MA** | **F. JIA** | **F. Controls** | **P** | **FDR** | **OR** | **95% CI** |  | **Chr** | **SNP** | **Position** | **MA** | **F. JIA** | **F. Controls** | **P** | **FDR** | **OR** | **95% CI** |
| 6 | rs28421666 | 32592737 | G | 0.17 | 0.05 | 8.88E-12 | 5.23E-06 | 4.32 | 2.84-6.58 |  | 6 | rs28421666 | 32592737 | G | 0.16 | 0.05 | 4.66E-08 | 2.44E-02 | 4.3 | 2.55-7.26 |
| 6 | rs9272105 | 32599999 | G | 0.26 | 0.45 | 1.09E-08 | 2.00E-03 | 0.44 | 0.33-0.58 |  | 6 | rs2071550 | 32730940 | A | 0.54 | 0.34 | 8.28E-08 | 2.44E-02 | 2.6 | 1.83-3.68 |
| 6 | rs2395148 | 32321554 | A | 0.13 | 0.043 | 1.27E-08 | 2.00E-03 | 3.62 | 2.32-5.63 |  | 6 | rs3763313 | 32376471 | C | 0.35 | 0.2 | 1.02E-06 | 1.51E-01 | 2.68 | 1.81-3.98 |
| 6 | rs9501173 | 32279902 | A | 0.17 | 0.06 | 1.32E-08 | 2.00E-03 | 3.15 | 2.12-4.69 |  | 6 | rs9469220 | 32658310 | A | 0.64 | 0.44 | 1.23E-06 | 1.51E-01 | 2.41 | 1.69-3.44 |
| 6 | rs3749967 | 32283844 | G | 0.17 | 0.06 | 1.32E-08 | 2.00E-03 | 3.15 | 2.12-4.69 |  | 2 | rs1250250 | 216289343 | A | 0.41 | 0.23 | 1.80E-06 | 1.51E-01 | 2.46 | 1.7-3.56 |
| 6 | rs9273012 | 32611641 | G | 0.43 | 0.26 | 2.51E-08 | 2.00E-03 | 2.18 | 1.66-2.86 |  | 2 | rs7588830 | 216277672 | A | 0.41 | 0.23 | 1.93E-06 | 1.51E-01 | 2.45 | 1.7-3.55 |
| 6 | rs9469099 | 32308908 | A | 0.13 | 0.04 | 3.02E-08 | 3.00E-03 | 3.48 | 2.24-5.4 |  | 2 | rs1250238 | 216294663 | A | 0.39 | 0.22 | 2.03E-06 | 1.51E-01 | 2.51 | 1.72-3.67 |
| 6 | rs9272219 | 32602269 | A | 0.43 | 0.26 | 3.40E-08 | 3.00E-03 | 2.15 | 1.64-2.83 |  | 10 | rs10827290 | 34098326 | A | 0.25 | 0.45 | 2.12E-06 | 1.51E-01 | 0.4 | 0.28-0.59 |
| 6 | rs17576984 | 32212985 | A | 0.22 | 0.1 | 7.75E-08 | 5.00E-03 | 2.57 | 1.82-3.63 |  | 2 | rs17517509 | 216284774 | A | 0.41 | 0.23 | 2.30E-06 | 1.51E-01 | 2.44 | 1.68-3.53 |
| 6 | rs7765379 | 32680928 | C | 0.24 | 0.12 | 1.63E-07 | 9.00E-03 | 2.37 | 1.71-3.26 |  | 13 | rs3803221 | 25241724 | A | 0.12 | 0.04 | 3.75E-06 | 2.21E-01 | 3.94 | 2.2-7.04 |

|  |  | **Systemic (n=23)** | | | | | | |  |  |  |  |  |  | **Enthesitis-related (n= 23)** | | | | |  |
| --- | --- | --- | --- | --- | --- | --- | --- | --- | --- | --- | --- | --- | --- | --- | --- | --- | --- | --- | --- | --- |
| **Chr** | **SNP** | **Position** | **MA** | **F. JIA** | **F. Controls** | **P** | **FDR** | **OR** | **95% CI** |  | **Chr** | **SNP** | **Position** | **MA** | **F. JIA** | **F. Controls** | **P** | **FDR** | **OR** | **95% CI** |
| 7 | rs3926927 | 105858211 | A | 0.39 | 0.1 | 7.87E-08 | 3.34E-02 | 9.78 | 4.25-22.47 |  | 6 | rs17206680 | 31379752 | A | 0.3 | 0.07 | 4.05E-07 | 2.35E-01 | 8.86 | 3.81-20.59 |
| 7 | rs10245392 | 105843081 | G | 0.39 | 0.1 | 1.15E-07 | 3.34E-02 | 9.59 | 4.16-22.12 |  | 6 | rs4418214 | 31391401 | G | 0.35 | 0.1 | 9.97E-07 | 2.71E-01 | 7.85 | 3.44-17.91 |
| 19 | rs11672396 | 16718713 | G | 0.35 | 0.1 | 5.86E-06 | 9.92E-01 | 5 | 2.49-10.03 |  | 14 | rs8015401 | 86243914 | G | 0.26 | 0.06 | 1.40E-06 | 2.71E-01 | 8.3 | 3.51-19.62 |
| 11 | rs10836470 | 4707571 | A | 0.39 | 0.13 | 7.51E-06 | 9.92E-01 | 4.73 | 2.4-9.35 |  | 2 | rs4145348 | 192047465 | A | 0.37 | 0.11 | 2.51E-06 | 2.88E-01 | 6.04 | 2.86-12.76 |
| 9 | rs1889283 | 82965051 | A | 0.39 | 0.14 | 1.09E-05 | 9.92E-01 | 5.16 | 2.48-10.73 |  | 1 | rs6688757 | 77550946 | C | 0.24 | 0.04 | 4.11E-06 | 2.88E-01 | 7.78 | 3.25-18.61 |
| 1 | rs7526119 | 14850351 | A | 0.5 | 0.24 | 1.46E-05 | 9.92E-01 | 4.48 | 2.27-8.82 |  | 6 | rs9266845 | 31384792 | G | 0.54 | 0.24 | 4.46E-06 | 2.88E-01 | 5.44 | 2.64-11.22 |
| 3 | rs1375823 | 22618061 | A | 0.44 | 0.2 | 1.88E-05 | 9.92E-01 | 4.29 | 2.2-8.34 |  | 6 | rs1131904 | 31383071 | G | 0.54 | 0.24 | 4.59E-06 | 2.88E-01 | 5.42 | 2.63-11.16 |
| 20 | rs7261002 | 1002656 | A | 0.44 | 0.17 | 1.94E-05 | 9.92E-01 | 4.56 | 2.27-9.14 |  | 6 | rs9295993 | 31388595 | G | 0.54 | 0.24 | 4.93E-06 | 2.88E-01 | 5.43 | 2.63-11.21 |
| 18 | rs7232133 | 42899840 | A | 0.52 | 0.26 | 2.41E-05 | 9.92E-01 | 4.28 | 2.18-8.41 |  | 6 | rs6932236 | 31383979 | G | 0.54 | 0.24 | 5.02E-06 | 2.88E-01 | 5.42 | 2.62-11.2 |
| 1 | rs16836356 | 238277625 | G | 0.17 | 0.04 | 2.46E-05 | 9.92E-01 | 6.75 | 2.78-16.4 |  | 19 | rs4805656 | 31724380 | A | 0.48 | 0.18 | 5.77E-06 | 2.88E-01 | 5.02 | 2.5-10.07 |

|  |  |  |  | **Psoriatic arthritis (n=18)** | | | | |  |  |  |  |  |  | **RF–positive polyarthritis (n=13)** | | | | |  |
| --- | --- | --- | --- | --- | --- | --- | --- | --- | --- | --- | --- | --- | --- | --- | --- | --- | --- | --- | --- | --- |
| **Chr** | **SNP** | **Position** | **MA** | **F. JIA** | **F. Controls** | **P** | **FDR** | **OR** | **95% CI** |  | **Chr** | **SNP** | **Position** | **MA** | **F. JIA** | **F. Controls** | **P** | **FDR** | **OR** | **95% CI** |
| 12 | rs7979218 | 46145212 | A | 0.14 | 0.01 | 1.48E-06 | 8.43E-01 | 21 | 4.25-22.47 |  | 18 | rs17083852 | 68848379 | G | 0.19 | 0.03 | 1.00E-04 | 9.12E-01 | 11.06 | 4.25-22.47 |
| 15 | rs1906401 | 53905646 | G | 0.5 | 0.16 | 4.27E-06 | 8.43E-01 | 6.3 | 4.16-22.12 |  | 2 | rs12714380 | 3489932 | G | 0.42 | 0.11 | 1.00E-04 | 9.12E-01 | 5.19 | 4.16-22.12 |
| 14 | rs11851709 | 99377959 | G | 0.19 | 0.02 | 5.95E-06 | 8.43E-01 | 12 | 2.49-10.03 |  | 7 | rs4144066 | 48540424 | G | 0.5 | 0.16 | 2.00E-04 | 9.12E-01 | 5.22 | 2.49-10.03 |
| 3 | rs16831329 | 160378752 | G | 0.17 | 0.02 | 6.78E-06 | 8.43E-01 | 15 | 2.4-9.35 |  | 2 | rs12994580 | 3472358 | A | 0.38 | 0.1 | 3.00E-04 | 9.12E-01 | 4.82 | 2.4-9.35 |
| 10 | rs4746841 | 71064512 | A | 0.31 | 0.07 | 9.21E-06 | 8.43E-01 | 6.8 | 2.48-10.73 |  | 2 | rs10188054 | 3479959 | G | 0.38 | 0.1 | 4.00E-04 | 9.12E-01 | 4.67 | 2.48-10.73 |
| 5 | rs17066578 | 165704020 | C | 0.17 | 0.03 | 1.18E-05 | 8.43E-01 | 11 | 2.27-8.82 |  | 15 | rs4778700 | 79787835 | A | 0.54 | 0.22 | 4.00E-04 | 9.12E-01 | 5.06 | 2.27-8.82 |
| 2 | rs2165440 | 130007996 | G | 0.14 | 0.02 | 1.22E-05 | 8.43E-01 | 15 | 2.2-8.34 |  | 12 | rs10878372 | 40716694 | G | 0.5 | 0.18 | 4.00E-04 | 9.12E-01 | 4.97 | 2.2-8.34 |
| 4 | rs11940750 | 183182681 | A | 0.28 | 0.06 | 1.25E-05 | 8.43E-01 | 7.5 | 2.27-9.14 |  | 2 | rs17801488 | 46481505 | A | 0.42 | 0.15 | 4.00E-04 | 9.12E-01 | 5.73 | 2.27-9.14 |
| 7 | rs1811248 | 28421036 | C | 0.71 | 0.32 | 1.39E-05 | 8.43E-01 | 6.6 | 2.18-8.41 |  | 14 | rs4898690 | 51848711 | A | 0.15 | 0.02 | 4.00E-04 | 9.12E-01 | 10.58 | 2.18-8.41 |
| 21 | rs9975129 | 33774784 | G | 0.33 | 0.1 | 1.47E-05 | 8.43E-01 | 6 | 2.78-16.4 |  | 8 | rs10099194 | 132145040 | G | 0.35 | 0.1 | 5.00E-04 | 9.12E-01 | 4.96 | 2.78-16.4 |

**Supplementary Table 3**. Expanded results of association tests of classical HLA alleles to JIA (n=329) compared to controls (n=748).

| **HLA** | **OR** | **95% CI** | **P** | **FDR** |
| --- | --- | --- | --- | --- |
| *DRB1*08:01* | 3.95 | 2.75-5.67 | 8.75E-14 | 1.40E-10 |
| *DQA1*04* | 3.66 | 2.57-5.21 | 5.28E-13 | 5.26E-10 |
| *DQA1*04:01* | 3.66 | 2.57-5.21 | 5.28E-13 | 5.26E-10 |
| *DRB1*08* | 3.55 | 2.51-5.02 | 7.92E-13 | 5.75E-10 |
| *DQB1*04* | 3.58 | 2.51-5.1 | 1.88E-12 | 6.26E-10 |
| *DQB1*04:02* | 3.58 | 2.51-5.1 | 1.88E-12 | 6.26E-10 |
| *DRB1*11* | 2.05 | 1.45-2.88 | 4.37E-05 | 1.10E-03 |
| *B*2705* | 2.03 | 1.44-2.85 | 5.04E-05 | 1.20E-03 |
| *B*27* | 1.95 | 1.39-2.73 | 9.71E-05 | 2.20E-03 |
| *DRB1*11:01* | 2.18 | 1.46-3.27 | 1.00E-04 | 2.90E-03 |
| *DQB1*02:02* | 0.32 | 0.17-0.59 | 3.00E-04 | 5.20E-03 |
| *DRB1*04* | 0.6 | 0.45-0.8 | 5.00E-04 | 7.10E-03 |
| *DPB1*02:01* | 1.56 | 1.21-2.02 | 6.00E-04 | 7.90E-03 |
| *DRB1*15* | 0.62 | 0.46-0.82 | 1.00E-03 | 1.07E-02 |
| *DRB1*15:01* | 0.62 | 0.46-0.83 | 1.30E-03 | 1.32E-02 |
| *DQB1*03:02* | 0.57 | 0.4-0.8 | 1.40E-03 | 1.35E-02 |
| *DQA1*03* | 0.65 | 0.49-0.84 | 1.40E-03 | 1.38E-02 |
| *DQA1*03:01* | 0.65 | 0.49-0.84 | 1.40E-03 | 1.38E-02 |
| *DRB1*04:01* | 0.56 | 0.39-0.8 | 1.50E-03 | 1.47E-02 |
| *DPB1*02* | 1.51 | 1.17-1.94 | 1.60E-03 | 1.49E-02 |
| *DQB1*06:02* | 0.62 | 0.46-0.84 | 1.90E-03 | 1.71E-02 |
| *A*0201* | 1.35 | 1.11-1.65 | 2.70E-03 | 2.19E-02 |
| *DQB1*02* | 0.64 | 0.48-0.86 | 2.70E-03 | 2.20E-02 |
| *A*02* | 1.34 | 1.1-1.63 | 4.00E-03 | 2.83E-02 |
| *DRB1*07* | 0.55 | 0.36-0.83 | 4.50E-03 | 3.00E-02 |
| *DRB1*07:01* | 0.55 | 0.36-0.83 | 4.50E-03 | 3.00E-02 |
| *DQA1*02* | 0.55 | 0.36-0.83 | 4.50E-03 | 3.00E-02 |
| *DQA1*02:01* | 0.55 | 0.36-0.83 | 4.50E-03 | 3.00E-02 |
| *A*03* | 0.69 | 0.52-0.91 | 9.70E-03 | 5.31E-02 |

**Supplementary Table 4**. Results of association tests of classical HLA alleles to JIA ILAR subtypes. AF: Allele Frequency

|  | **Oligoarthritis** **(n=157)** | | | | |  |
| --- | --- | --- | --- | --- | --- | --- |
| **HLA** | **AF JIA** | **AF Controls** | **OR** | **95% CI** | **P** | **FDR** |
| *DRB1*08:01* | 0.16 | 0.04 | 4.98 | 3.22-7.7 | 5.60E-13 | 9.31E-10 |
| *DRB1*08* | 0.16 | 0.05 | 4.41 | 2.9-6.71 | 3.98E-12 | 1.50E-09 |
| *DQA1*04* | 0.17 | 0.05 | 4.67 | 3.05-7.15 | 1.29E-12 | 1.34E-09 |
| *DQA1*04:01* | 0.17 | 0.05 | 4.67 | 3.05-7.15 | 1.29E-12 | 1.34E-09 |
| *DQB1*04* | 0.16 | 0.05 | 4.58 | 2.98-7.02 | 3.26E-12 | 1.43E-09 |
| *DQB1*04:02* | 0.16 | 0.05 | 4.58 | 2.98-7.02 | 3.26E-12 | 1.43E-09 |
| *DRB1*11* | 0.15 | 0.06 | 2.61 | 1.72-3.95 | 5.91E-06 | 2.80E-04 |
| *DRB1*11:01* | 0.09 | 0.04 | 2.55 | 1.56-4.17 | 1.97E-04 | 6.03E-03 |
| *DPB1*02:01* | 0.24 | 0.15 | 1.82 | 1.32-2.51 | 2.45E-04 | 0.006.93 |
| *DRB1*07:01* | 0.02 | 0.08 | 0.23 | 0.1-0.52 | 4.14E-04 | 9.33E-03 |
| *DRB1*07* | 0.02 | 0.08 | 0.23 | 0.1-0.52 | 4.14E-04 | 9.33E-03 |
| *DQA1*02* | 0.02 | 0.08 | 0.23 | 0.1-0.52 | 4.14E-04 | 9.33E-03 |
| *DPB1*02* | 0.24 | 0.15 | 1.78 | 1.29-2.45 | 4.18E-04 | 9.36E-03 |
| *DQA1*02:01* | 0.02 | 0.08 | 0.23 | 0.1-0.52 | 4.14E-04 | 9.33E-03 |
| *DRB1*04* | 0.09 | 0.17 | 0.52 | 0.34-0.78 | 1.57E-03 | 2.29E-02 |
| *DQB1*02* | 0.01 | 0.17 | 0.51 | 0.34-0.78 | 1.77E-03 | 2.37E-02 |
| *DQB1*03:02* | 0.06 | 0.12 | 0.44 | 0.27-0.74 | 1.79E-03 | 2.37E-02 |
| *DRB1*11:04* | 0.05 | 0.01 | 3.07 | 1.5-6.25 | 2.06E-03 | 2.63E-02 |
| *DQB1*02:02* | 0.01 | 0.05 | 0.16 | 0.05-0.52 | 2.20E-03 | 2.76E-02 |
| *B*27:05* | 0.11 | 0.06 | 1.94 | 1.25-3.02 | 3.32E-03 | 3.46E-02 |
| *A*02* | 0.44 | 0.36 | 1.48 | 1.14-1.92 | 3.34E-03 | 3.46E-02 |
| *DQA1*03:01* | 0.12 | 0.19 | 0.57 | 0.4-0.84 | 3.78E-03 | 3.63E-02 |
| *DQA1*03* | 0.12 | 0.19 | 0.57 | 0.4-0.84 | 3.78E-03 | 3.63E-02 |
| *A*02:01* | 0.43 | 0.36 | 1.47 | 1.13-1.9 | 4.12E-03 | 3.80E-02 |
| *B*40* | 0.13 | 0.08 | 1.76 | 1.19-2.59 | 4.73E-03 | 4.23E-02 |
| *C*06:02* | 0.03 | 0.08 | 0.38 | 0.19-0.74 | 4.90E-03 | 4.32E-02 |
| *C*06* | 0.03 | 0.08 | 0.38 | 0.19-0.74 | 4.90E-03 | 4.32E-02 |
| *B*27* | 0.11 | 0.07 | 1.86 | 1.21-2.88 | 5.16E-03 | 4.44E-02 |
| *A*03* | 0.1 | 0.16 | 0.57 | 0.38-0.85 | 6.04E-03 | 4.89E-02 |

|  | **RF-negative polyarthritis (n=91)** | | | | | |  |
| --- | --- | --- | --- | --- | --- | --- | --- |
| **HLA** | **AF JIA** | **AF Controls** | **OR** | **95% CI** | **P** | **FDR** |  |
| *DRB1*08* | 0.16 | 0.05 | 4.32 | 2.56-7.29 | 4.33E-08 | 2.97E-05 | |
| *DRB1*08:01* | 0.14 | 0.04 | 4.5 | 2.62-7.72 | 5.03E-08 | 2.97E-05 |  |
| *DQA1*04* | 0.15 | 0.05 | 4.16 | 2.45-7.09 | 1.53E-07 | 4.52E-05 |  |
| *DQA1*04:01* | 0.15 | 0.05 | 4.16 | 2.45-7.09 | 1.53E-07 | 4.52E-05 |  |
| *DQB1*04* | 0.14 | 0.05 | 3.95 | 2.31-6.75 | 5.08E-07 | 9.33E-05 |  |
| *DQB1*04:02* | 0.14 | 0.05 | 3.95 | 2.31-6.75 | 5.08E-07 | 9.33E-05 |  |
| *DRB1*04* | 0.04 | 0.17 | 0.23 | 0.11-0.49 | 1.50E-04 | 5.42E-03 |  |
| *DQA1*03* | 0.07 | 0.19 | 0.37 | 0.21-0.67 | 9.00E-04 | 1.97E-02 |  |
| *DQA1*03:01* | 0.07 | 0.19 | 0.37 | 0.21-0.67 | 9.00E-04 | 1.97E-02 |  |
| *DQB1*03:02* | 0.03 | 0.12 | 0.22 | 0.09-0.55 | 1.00E-03 | 2.02E-02 |  |
| *DRB1*04:01* | 0.02 | 0.12 | 0.16 | 0.05-0.51 | 1.77E-03 | 3.36E-02 |  |

| **Systemic (n=23)** | | | | | | |
| --- | --- | --- | --- | --- | --- | --- |
| **HLA** | **AF JIA** | **AF Controls** | **OR** | **95% CI** | **P** | **FDR** |
| *DRB1*11:01* | 0.17 | 0.04 | 5.73 | 2.21-14.9 | 3.40E-04 | 9.50E-01 |
| *DRB1*11* | 0.2 | 0.06 | 3.37 | 1.41-8.04 | 6.26E-03 | 9.50E-01 |
| *C*07:04* | 0.07 | 0.01 | 4.64 | 1.3-16.65 | 1.84E-02 | 9.50E-01 |

|  | **Enthesitis-related (n= 23)** | | | |  |  |
| --- | --- | --- | --- | --- | --- | --- |
| **HLA** | **AF JIA** | **AF Controls** | **OR** | **95% CI** | **P** | **FDR** |
| *B*27:05* | 0.3 | 0.06 | 9.92 | 4.27-23.04 | 9.52E-08 | 2.86E-04 |
| *B*27* | 0.3 | 0.06 | 9.02 | 3.9-20.9 | 2.87E-07 | 2.86E-04 |
| *A*26* | 0.13 | 0.03 | 6.16 | 2.09-18.2 | 1.00E-03 | 8.45E-02 |
| *A*26:01* | 0.13 | 0.03 | 6.16 | 2.09-18.2 | 1.00E-03 | 8.45E-02 |

|  | **Psoriatic (n=18)** | | | |  |  |
| --- | --- | --- | --- | --- | --- | --- |
| **HLA** | **AF JIA** | **AF Controls** | **OR** | **95% CI** | **P** | **FDR** |
| *B*57* | 0.1 | 0.03 | 7.13 | 2-25.43 | 2.45E-03 | 3.19E-01 |
| *B*57:01* | 0.1 | 0.03 | 7.13 | 2-25.43 | 2.45E-03 | 3.19E-01 |
| *B*38* | 0.1 | 0 | 10.8 | 1.97-58.72 | 6.09E-03 | 3.88E-01 |
| *B*38:01* | 0.1 | 0 | 10.8 | 1.97-58.72 | 6.09E-03 | 3.88E-01 |

|  | **RF–positive polyarthritis (n=13)** | | | |  |  |
| --- | --- | --- | --- | --- | --- | --- |
| **HLA** | **AF JIA** | **AF Controls** | **OR** | **95% CI** | **P** | **FDR** |
| *A*31* | 0.12 | 0.03 | 5.22 | 1.31-20.83 | 1.91E-02 | 0.595 |
| *A*31:01* | 0.12 | 0.03 | 5.22 | 1.31-20.83 | 1.91E-02 | 0.595 |
| *DRB1*04* | 0.35 | 0.03 | 2.79 | 1.16-6.71 | 2.15E-02 | 0.595 |


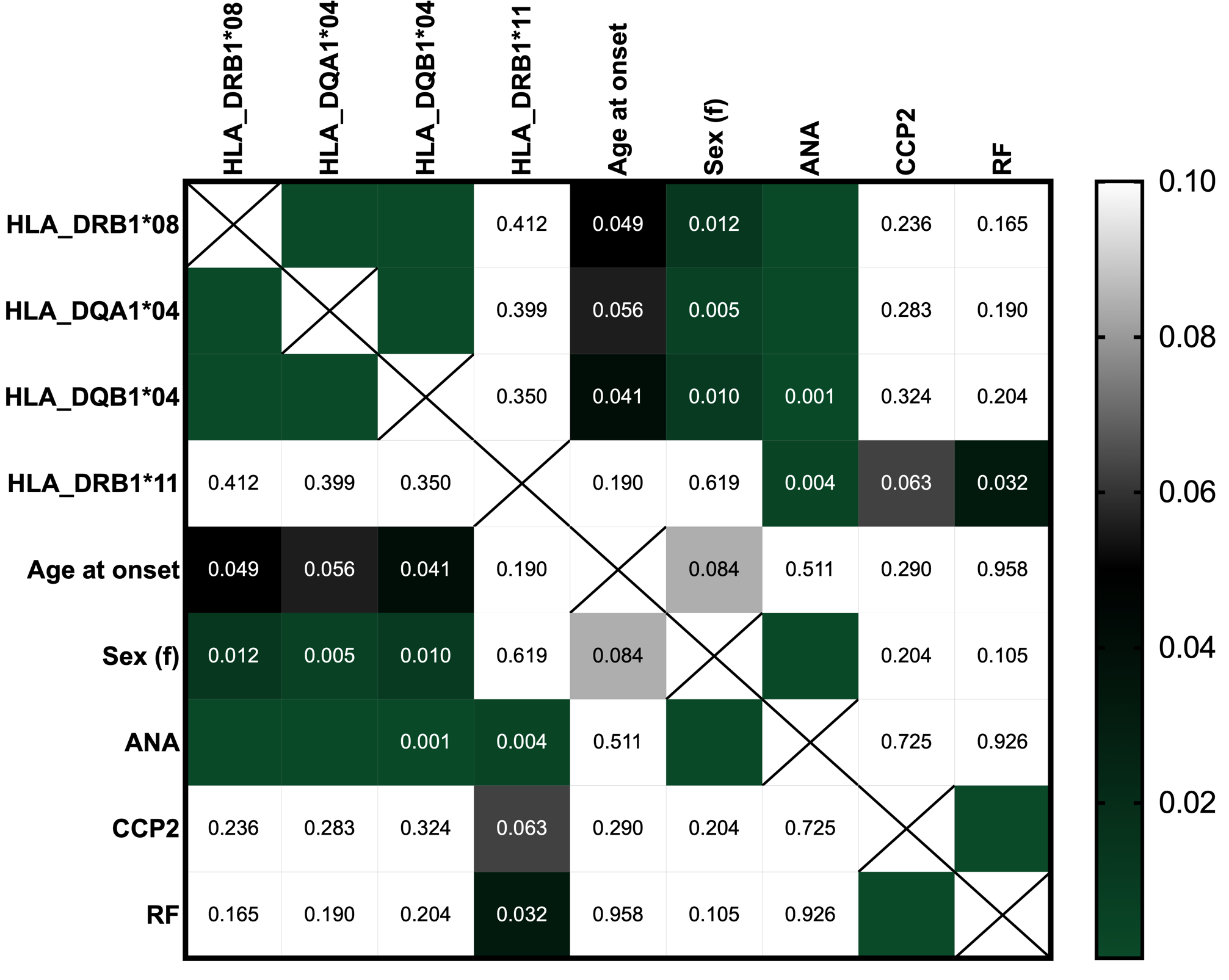

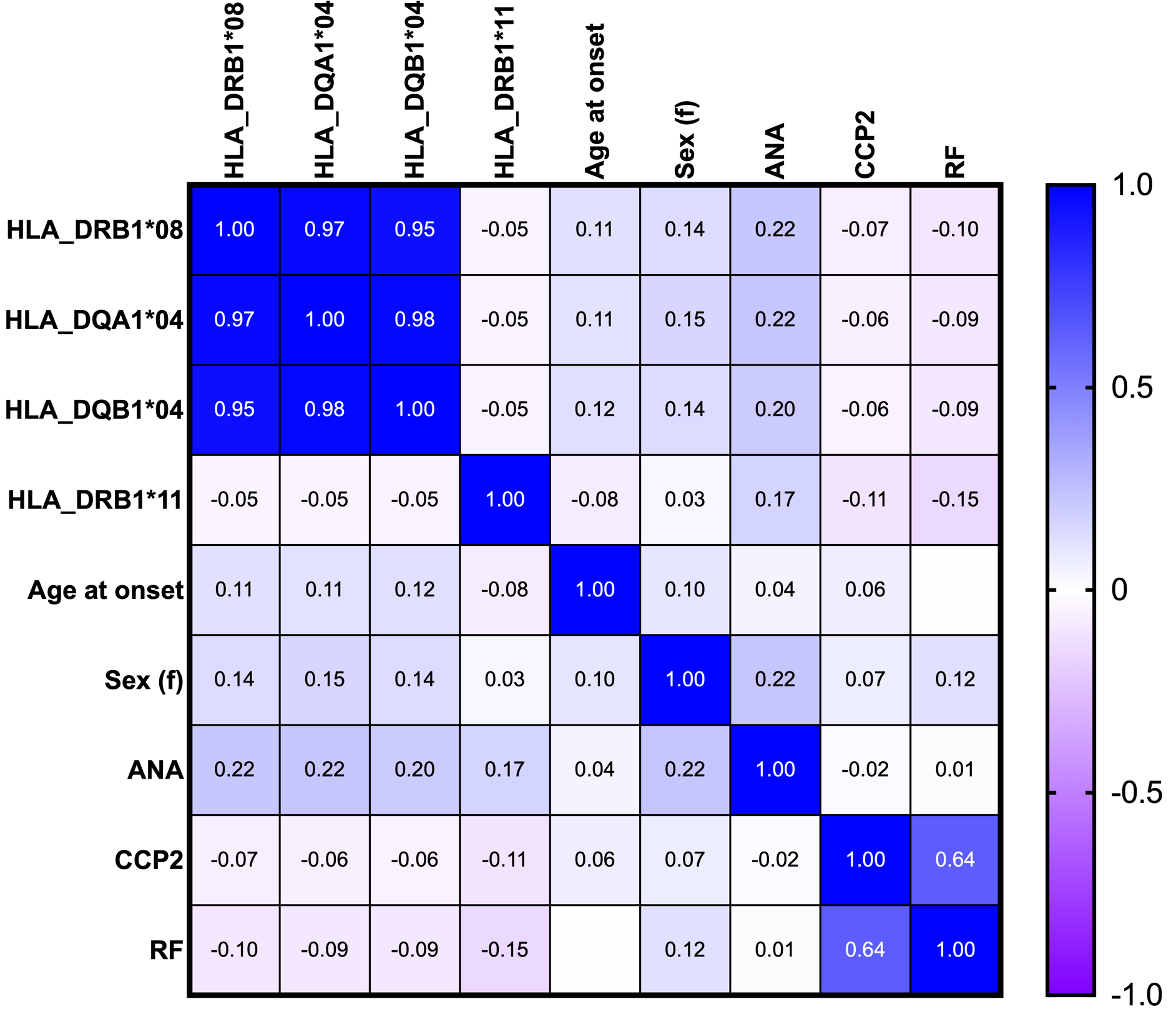


**A.** **B.**

**Supplementary Figure 2. A**. Heatmaps of correlation analysis between associated HLA alleles that depicts Phi coefficient r ranging from -1.0 to 1.0. and other patient related parameters. **B.** Depicts P values associated with the correlation analysis. Sex (females), Age at onset (month) and measured autoantibodies, ANA, ACPA and RF.

**Supplementary Table 5**. Expanded results of association tests of classical *HLA* alleles to ANA-positive JIA (n=144) compared to controls (n=748). AF: Allele Frequency

| **HLA** | **AF** | **AF** | **OR** | **95% CI** | **P** | **FDR** |
| --- | --- | --- | --- | --- | --- | --- |
|  | **JIA** | **Controls** |  |  |  |  |
| *DRB1*08* | 0.21 | 0.05 | 6.42 | 4.2-9.9 | 1.19E-17 | 8.97E-15 |
| *DRB1*08:01* | 0.19 | 0.04 | 7.03 | 4.6-11 | 4.00E-18 | 6.65E-15 |
| *DQA1*04* | 0.2 | 0.05 | 6.63 | 4.4-10.3 | 9.44E-18 | 8.97E-15 |
| *DQA1*04:01* | 0.2 | 0.05 | 6.63 | 4.4-10.3 | 9.44E-18 | 8.97E-15 |
| *DQB1*04* | 0.19 | 0.05 | 6.34 | 4.2-9.8 | 8.25E-17 | 2.86E-14 |
| *DQB1*04:02* | 0.19 | 0.05 | 6.34 | 4.2-9.8 | 8.25E-17 | 2.86E-14 |
| *DRB1*11* | 0.16 | 0.06 | 2.84 | 1.9-4.4 | 1.59E-06 | 6.61E-05 |
| *DRB1*11:01* | 0.1 | 0.04 | 2.83 | 1.8-4.7 | 3.57E-05 | 7.35E-04 |
| *DPB1*02* | 0.27 | 0.15 | 2.15 | 1.6-3 | 4.29E-06 | 1.29E-04 |
| *DPB1*02:01* | 0.27 | 0.15 | 2.19 | 1.6-3.1 | 2.35E-06 | 9.01E-05 |
| *DQB1*06* | 0.24 | 0.3 | 0.4 | 0.3-0.7 | 2.78E-04 | 3.62E-03 |
| *DQB1*06:02* | 0.07 | 0.16 | 0.4 | 0.3-0.7 | 2.78E-04 | 3.62E-03 |
| *B*27* | 0.12 | 0.07 | 2.22 | 1.5-3.5 | 3.43E-04 | 4.17E-03 |
| *B*2705* | 0.11 | 0.06 | 2.22 | 1.5-3.5 | 4.83E-04 | 5.07E-03 |
| *A*02* | 0.46 | 0.36 | 1.62 | 1.3-2.2 | 4.31E-04 | 4.74E-03 |
| *A*0201* | 0.45 | 0.36 | 1.63 | 1.3-2.2 | 3.50E-04 | 4.20E-03 |
| *DRB1*15* | 0.07 | 0.16 | 0.45 | 0.3-0.8 | 5.67E-04 | 5.77E-03 |
| *DRB1*15:01* | 0.07 | 0.16 | 0.44 | 0.3-0.8 | 7.14E-04 | 6.76E-03 |
| *DRB1*07* | 0.02 | 0.08 | 0.21 | 0.1-0.6 | 6.84E-04 | 6.54E-03 |
| *DRB1*07:01* | 0.02 | 0.08 | 0.21 | 0.1-0.6 | 6.84E-04 | 6.54E-03 |
| *DRB1*04* | 0.09 | 0.17 | 0.37 | 0.3-0.7 | 1.15E-03 | 2.08E-02 |
| *DRB1*04:01* | 0.04 | 0.12 | 0.37 | 0.3-0.7 | 1.15E-03 | 9.70E-03 |
| *B*40* | 0.11 | 0.08 | 2.02 | 1.4-3.2 | 1.50E-03 | 1.42E-02 |
| *C*02* | 0.11 | 0.06 | 2.03 | 1.3-3.2 | 2.03E-03 | 1.48E-02 |
| *C*0202* | 0.11 | 0.06 | 2.03 | 1.3-3.2 | 2.03E-03 | 1.48E-02 |
| *DQA1*03* | 0.11 | 0.19 | 0.55 | 0.4-0.9 | 3.28E-03 | 2.09E-02 |
| *DQA1*03:01* | 0.11 | 0.19 | 0.55 | 0.4-0.9 | 3.28E-03 | 2.09E-02 |
| *DQB1*02* | 0.11 | 0.17 | 0.53 | 0.4-0.9 | 3.43E-03 | 2.16E-02 |
| *DQB1*02:02* | 0.01 | 0.05 | 0.12 | 0.1-0.5 | 3.25E-03 | 2.09E-02 |
| *C*06* | 0.03 | 0.08 | 0.35 | 0.2-0.8 | 5.74E-03 | 3.39E-02 |
| *C*06:02* | 0.03 | 0.08 | 0.35 | 0.2-0.8 | 5.74E-03 | 3.39E-02 |
| *A*03* | 0.1 | 0.16 | 0.55 | 0.4-0.9 | 6.20E-03 | 3.58E-02 |
| *DRB1*11:04* | 0.05 | 0.01 | 2.63 | 1.3-5.7 | 1.28E-02 | 6.30E-02 |
| *DQB1*0201* | 0.1 | 0.11 | 0.8 | 0.6-1.3 | 3.28E-01 | 5.45E-01 |


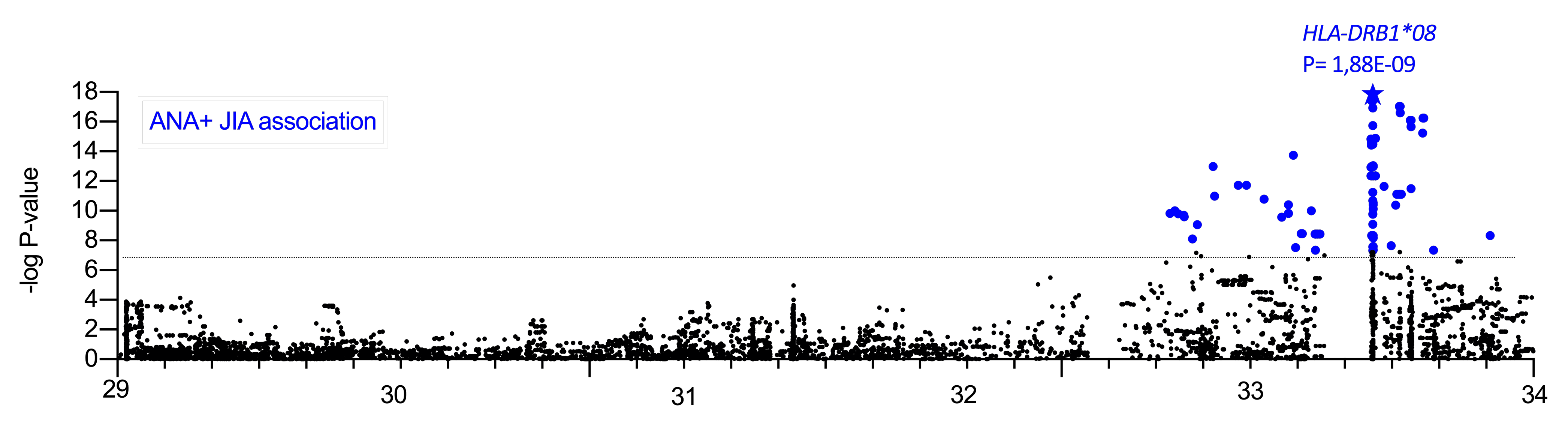


**A.**


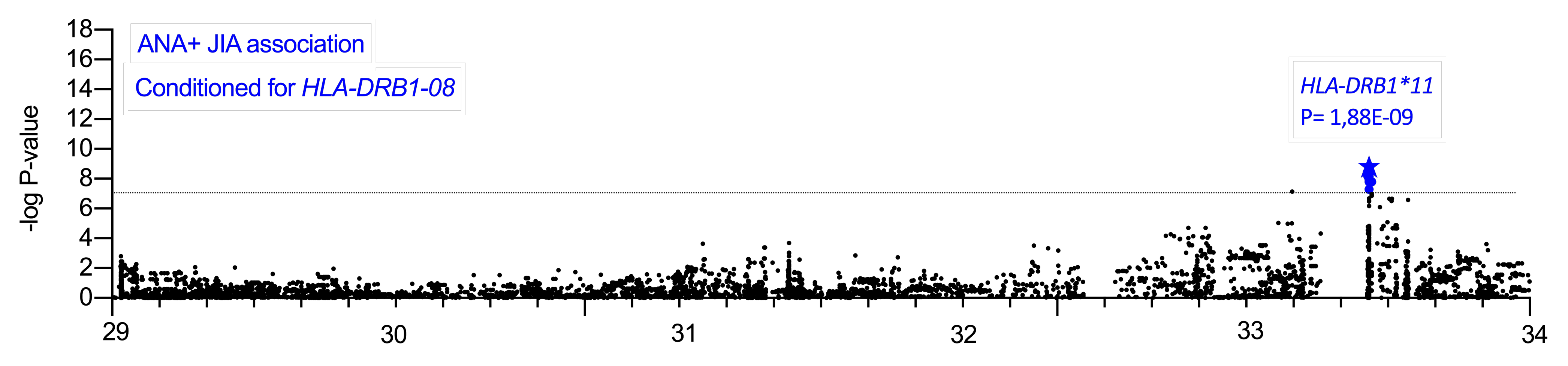


**B.**


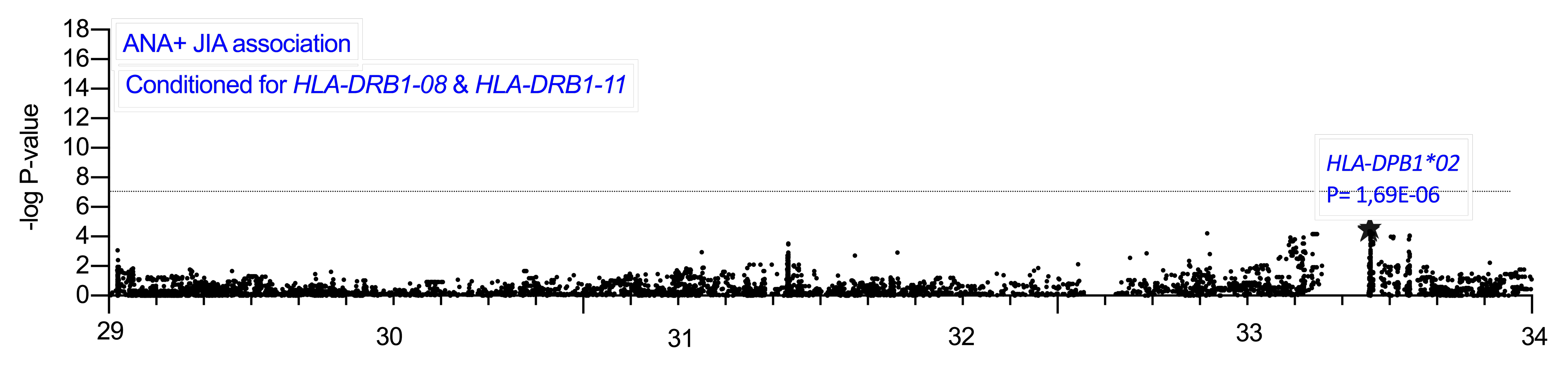


**C**.


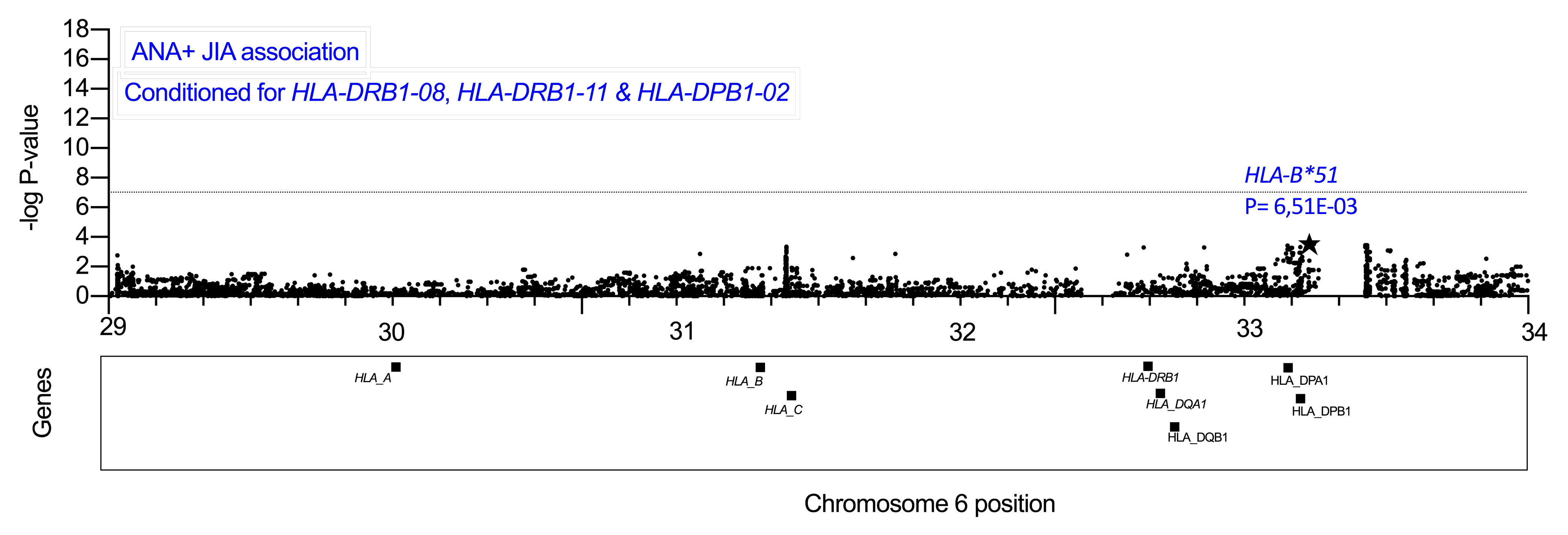


**D**.


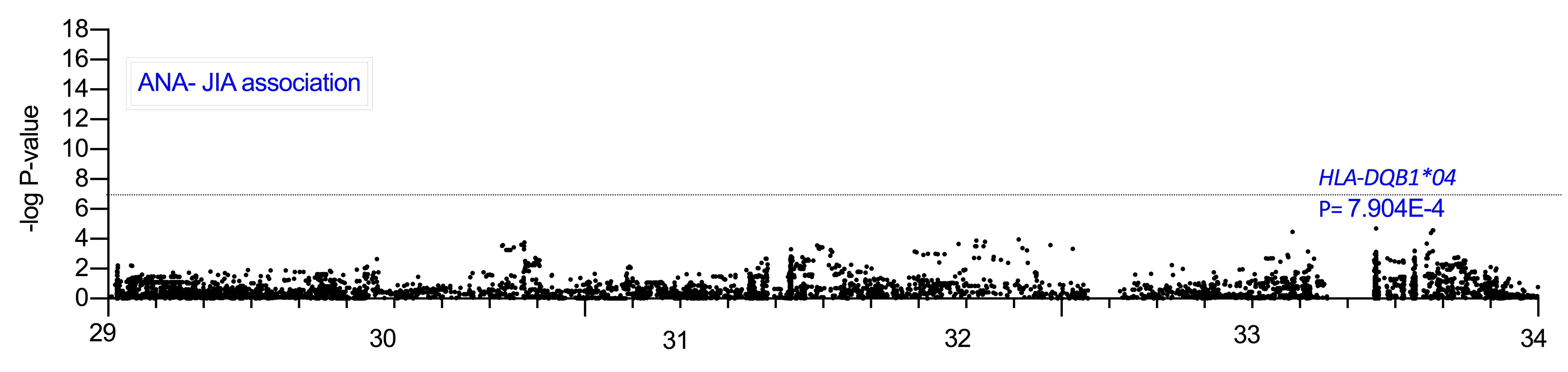


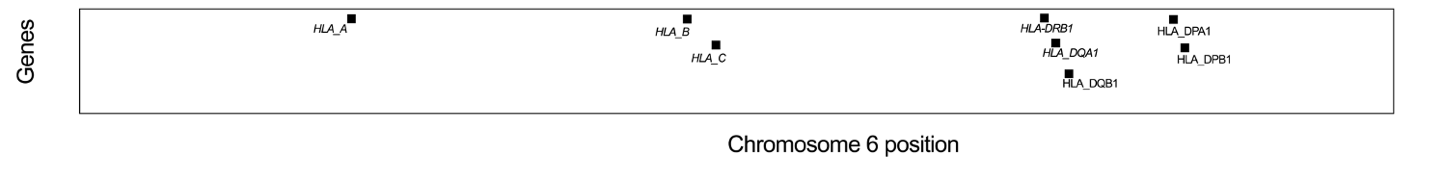


**E**.

**Supplementary Figure 3**. Association tests within the Major Histocompatibility Complex (MHC) for **A.** ANA-positive JIA (n= 144), **B-D.** subsequent conditional analyses to determine the independent effect of associated imputed HLA markers on ANA-positive JIA. E. ANA-negative JIA (n= 153) compared to controls (n=748). The genome-wide significance threshold (P < 5x10^-8, -log10(P) > 7.3) is indicated by the line. The star denotes the top hit.

**Supplementary Table 6**. Logistic Regression in 297 JIA patients having ANA as dependent variable.

| **Variable** | **B** | **SE** | **OR** | **95% CI** | **P value** |
| --- | --- | --- | --- | --- | --- |
| Intercept | -0.2 | 0.34 | 0.82 | 0.42- 1.6 | 0.5608 |
| *DRB1*08* | 0.79 | 0.29 | 2.2 | 1.26- 3.89 | 0.006 |
| *DRB1*11* | 0.77 | 0.3 | 2.17 | 1.2-3.97 | 0.0109 |
| Sex-females | 0.75 | 0.29 | 2.11 | 1.21-3.73 | 0.0091 |
| Age at onset | -0.01 | 0 | 0.99 | 0.99- 0.99 | <0.0001 |

B refers to the regression coefficient and SE refers to the standard error of the mean.
